# Temperature-Dependent Enzyme-Ligand Recognition Emerges from the Coupling of Protein Dynamics and Hydration

**DOI:** 10.1021/acsomega.6c09199

**Published:** 2026-10-21

**Authors:** Binny A. Rudani, Mona Sarter, Tilo Seydel, Simone Wiegand, Andreas M. Stadler

**Affiliations:** † Institute of Biological Information Processing IBI-4:Biomacromolecular Systems and Processes, 28334 Forschungszentrum Jülich GmbH, 52425 Jülich, Germany; ‡ ISIS Neutron and Muon Facility, STFC Rutherford Appleton Laboratory, Didcot OX11 0QX, U.K.; § 56053 Institut Laue-Langevin, 71 Avenue des Martyrs, CS 20156, 38042 Grenoble, Cedex 9, France; ∥ Department für Chemie—Institut für Licht und Materialien, Universität zu Köln, 50939 Cologne, Germany; ⊥ Jülich Centre for Neutron Science JCNS-1: Neutron Scattering and Soft Matter, Forschungszentrum Jülich GmbH, 52425 Jülich, Germany; # Institute of Physical Chemistry, RWTH Aachen University, Landoltweg 2, 52056 Aachen, Germany

## Abstract

Protein–ligand interactions are governed by a subtle interplay between binding thermodynamics, protein dynamics, and hydration. However, how these factors are coupled remains poorly understood, particularly for enzymes whose function depends on coordinated water rearrangements. Here, we investigate the binding of the fluorinated sulfonamide inhibitors 4-fluorobenzenesulfonamide (4FBS) and pentafluorobenzenesulfonamide (PFBS) to the zinc metalloenzyme bovine carbonic anhydrase (BCA) at 298 and 308 K. Binding thermodynamics were characterized by isothermal titration calorimetry (ITC), while quasi-elastic neutron scattering (QENS) was employed to probe ligand-induced changes in protein dynamics. ITC measurements in D_2_O were complemented by measurements in H_2_O over 288–318 K to assess temperature and solvent isotope effects. PFBS binds more strongly to BCA than 4FBS over the entire temperature range, whereas the thermodynamic parameters are similar in H_2_O and D_2_O. QENS-derived estimates indicate that binding of either ligand increases the conformational entropy of BCA. In contrast to the thermodynamic trends, however, the dynamic response depends strongly on the ligand: the diffusion coefficient associated with localized internal motions decreases with increasing temperature for the BCA-PFBS complex but increases for BCA-4FBS, indicating that the two ligands modulate the protein energy landscape in distinct ways. Despite their closely related structures, PFBS and 4FBS exhibit markedly different temperature-dependent effects on BCA dynamics. Our results show that subtle changes in ligand chemistry can reshape the balance between protein flexibility, hydration, and binding thermodynamics.

## Graphical abstract

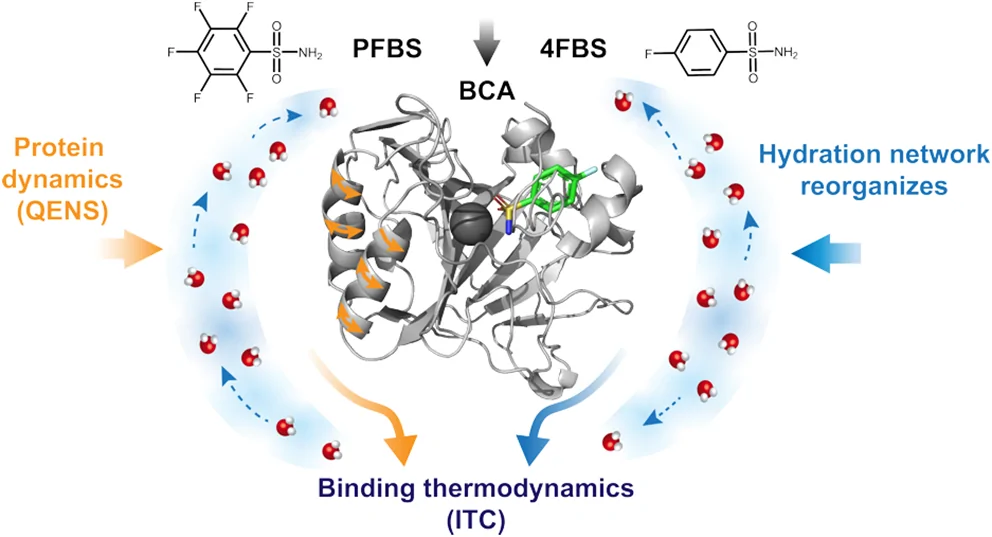

## Introduction

Carbonic anhydrases (CAs) are zinc-containing metalloenzymes and important therapeutic targets. Their inhibitors are widely used to treat glaucoma, acute mountain sickness, epilepsy, and other diseases, while selective inhibition of specific isoforms is actively explored for cancer therapy.^1–3^ However, how ligand binding modulates protein dynamics and how these changes contribute to binding thermodynamics remain poorly understood.

Unlike many protein–ligand systems, inhibitor binding to carbonic anhydrase involves not only changes in protein dynamics but also in a highly organized network of water molecules within the active site.^3–6^ Recent crystallographic studies showed that the exceptional catalytic efficiency of the enzyme relies on rapid rearrangements of this water network while the overall protein structure remains largely unchanged,^7^ consistent with earlier mechanistic studies.^8^ These findings suggest that enzyme function is governed by coupled protein–water dynamics, which makes it important to understand how inhibitor binding modifies both the protein motions and the active-site hydration network.

Therefore, understanding inhibitor binding to carbonic anhydrase requires identifying the molecular origins of the binding free energy. While the overall binding enthalpy and entropy can be determined experimentally, the microscopic origin of the entropy remains difficult to resolve. Increasing evidence indicates that hydration-shell reorganization and changes in protein conformational dynamics both contribute significantly to molecular recognition.^9–12^ These contributions are expected to be closely coupled for carbonic anhydrase, because ligand binding perturbs both the active-site water network and the dynamic properties of the protein.

Disentangling these contributions requires complementary experimental techniques. Isothermal titration calorimetry (ITC) quantifies the overall thermodynamics of binding, whereas quasi-elastic neutron scattering (QENS) probes ligand-induced changes in protein dynamics on the picosecond-to-nanosecond time scale. Together with previous thermal diffusion forced Rayleigh scattering (TDFRS) studies of hydration,^12–15^ these approaches provide a framework for relating binding thermodynamics to changes in protein dynamics and hydration.

Earlier applications of this experimental approach focused on the streptavidin–biotin complex,^12,13,15^ one of the strongest known noncovalent protein–ligand interactions. In the present work, we extend this methodology to bovine carbonic anhydrase (BCA), a more moderately binding enzyme system in which the interplay between protein dynamics and hydration is expected to play a central role.

Although ligand binding is often associated with reduced protein flexibility, this behavior is not universal.^16,17^ Frederick et al. demonstrated for calmodulin that different ligands can induce markedly different dynamical responses despite exhibiting similar binding affinities.^9^ While some ligands substantially rigidified the protein, others produced little change in flexibility. Moreover, they found a strong correlation between the protein conformational entropy and the total binding entropy measured calorimetrically, indicating that comparable binding affinities can arise from different balances of enthalpic and entropic contributions.

For carbonic anhydrase, NMR relaxation and hydrogen–deuterium exchange experiments have shown that ligand binding can increase the conformational entropy of the protein.^18,19^ Together with the observation that the average structure changes only minimally upon ligand binding,^7,8,20^ these studies indicate that the relevant information is contained in fast dynamics rather than static structures. Both NMR relaxation and QENS provide information on fast protein dynamics on the picosecond-to-nanosecond time scale. While NMR relaxation yields site-specific information on molecular motions through their effects on nuclear spin relaxation, QENS directly probes hydrogen-dominated atomic motions through the energy and momentum dependence of incoherent neutron scattering, providing complementary information on the average internal dynamics of the protein.

The present study investigates the binding of two fluorinated benzenesulfonamide inhibitors, 4-fluorobenzenesulfonamide (4FBS) and pentafluorobenzenesulfonamide (PFBS), to BCA. Previous TDFRS measurements showed that free BCA is slightly more hydrophilic than the corresponding protein–ligand complexes, whereas the BCA+PFBS and BCA+4FBS complexes exhibit nearly identical hydrophilicity and only a weak temperature dependence of the Soret coefficient.^14^ Earlier ITC measurements further indicated stronger binding of PFBS than 4FBS.^14,21^ To establish a consistent thermodynamic basis for comparison with neutron scattering, we repeated the ITC measurements in both H_2_O and D_2_O and performed complementary QENS measurements at 298 and 308 K.

By combining QENS with ITC, we investigate how the temperature dependence of inhibitor binding is related to changes in protein dynamics and hydration, thereby testing whether the coupled protein–water dynamics contribute to the thermodynamics of ligand binding.^11,22,23^

## Materials and Methods

### Sample Preparation

#### ITC

BCA (purity ≥95% as determined by SDS–PAGE) was purchased from Sigma-Aldrich. According to the supplier, the preparation consists predominantly of BCA II with minor amounts of BCA I and BCA IV. PFBS and 4FBS were also obtained from Sigma-Aldrich. The purity of 4FBS was specified as 98%, whereas PFBS was supplied as pro analysi (p.a.) grade product, indicating analytical reagent quality.

The concentration used for the ITC experiments with BCA and PFBS concentrations was 22 μM and 220 μM, respectively. For BCA+4FBS we increased enzyme and ligand concentrations to 42 μM and 420 μM, respectively. All the experiments were performed in 20 mM sodium phosphate (NaP) buffer in D_2_O and H_2_O. Titration to the appropriate pH of 7.4 was performed using DCl/NaOD or HCl/NaOH. The concentration of BCA was determined by UV absorbance with a NanoDrop 2000/2000c spectrophotometer using an extinction coefficient of *E*_1%_ = 19.^24^ Ligand stock solutions were prepared gravimetrically using an analytical balance (readability: ± 0.1 mg).

#### QENS

The same batches of BCA and ligands were used to prepare the solutions for the ITC and QENS experiments. All concentrations were determined by weighing the compounds. The BCA concentration for the QENS experiments was 1.68 mM, with the corresponding ligand concentrations added (see Table S1 in the SI). All the samples were prepared in 20 mM NaP buffer in D_2_O at pH of 7.4. For both systems, the ligand concentration was sufficiently high that the fraction of free protein was below 2·10^–4^ (see Section S7 in the SI). The pH adjustment and concentration determination were performed using the same procedures as those used for the ITC measurements.

To ensure that no exchange of the surface hydrogens of BCA with the D_2_O buffer occurred during the QENS experiments, the freely exchangeable hydrogen atoms of BCA were exchanged beforehand. For this purpose BCA was incubated in D_2_O for 24 h, before it was lyophilized. The resulting powder was then reconstituted in the appropriate buffer.

### Techniques and Data Reduction

#### ITC Measurements

Isothermal titration calorimetry (ITC) is widely used to characterize protein–ligand interactions because it directly determines the binding affinity together with the associated changes in enthalpy Δ*H*, changes in entropy ΔS and Gibbs free energy ΔG can be calculated.^25,26^ The method is based on the stepwise injection of one reagent into a calorimetric cell, and its principles have been described elsewhere.^25,27,28^ In this study, all measurements were performed using a MicroCal PEAQ ITC (Malvern Panalytical).

We performed measurements at two temperatures, 298 and 308 K for D_2_O. For H_2_O, we measured between 288 and 318 K in an interval of 10 K to get insight about its temperature dependence and solvent effects. For each experiment, ligand solution was added from a syringe into the sample cell loaded with BCA solution. A typical experiment consisted of 19 injections, 2 μL each. The time interval between injections was 2.5 min. Measurements were repeated twice at each temperature and later the ligand was titrated into buffer in order to subtract the background enthalpies. The integrated heat of each injection, after background subtraction, was analyzed using MicroCal PEAQ-ITC Analysis Software (Malvern Panalytical) and fitted by nonlinear regression to a single-site binding model. The binding stoichiometry *N*, dissociation constant *K*_d_, and binding enthalpy Δ*H* were obtained from the fits, with *N* remaining close to unity (average deviation of approximately 5%). The Gibbs free energy and entropy changes were determined using Δ*G* = *RT* ln *K*_d_ and Δ*S* = (Δ*H* – Δ*G*)/*T*, respectively.^29^ The reported thermodynamic parameters represent the average of two independent measurements at each temperature, and the associated uncertainties were estimated from the variation between replicate experiments.

#### QENS Measurements

QENS measurements were obtained from two different instruments, IN16B at the ILL and IRIS at ISIS—Neutron and Muon Source.^30–32^ IN16B was used in BATS mode with the Si(111) analyzer leading to a full-width at half-maximum (fwhm) energy resolution of Δ*E* = 3.5 μeV, while IRIS was used with the PG002 analyzer, which provided an energy resolution of Δ*E* = 17.5 μeV. The instrument resolution was measured on both instruments using an annular vanadium standard. All samples were measured at 298 K and at 308 K. The typical ILL/ISIS annular sample cans were used with a gap size of 0.3 mm/0.5 mm for BCA+4FBS (ILL) or 0.1 mm (ISIS). All measurements on IN16B were performed for 5–7 h, while on IRIS the samples were measured for 12 h each to account for the difference in neutron flux between the two instruments.

#### QENS Data Reduction and Analysis

The IN16B measurements were processed with the standard IN16B reduction script, while the IRIS data were reduced using the graphical user interface. All subsequent data processing was carried out with Mantid 6.12 ^33–35^ For the reduced data the buffer was subtracted from the sample using the following expression

1 $$S\left(q,\omega \right)=S{\left(q,\omega \right)}_{\mathrm{sample}}-\left(1-\Phi \right)\cdot f\cdot S{\left(q,\omega \right)}_{\mathrm{buffer}}$$

Here Φ = *cv* is the partial volume fraction of the protein and *f* = *T*_protein_/*T*_buffer_ with the transmissions *T*_protein_ and *T*_buffer_ of the protein solution and the D_2_O buffer, respectively. This resulted in a correction factor of (1 – Φ) · *f* = 0.92592.

According to Sarter et al.,^36^ the region in which the coherent contribution to the scattered signal can no longer be considered negligible was determined. Details can be found in Section S3 in the SI. For further QENS data analysis, a *q*-range where incoherent scattering is dominant was determined to be 0.44 Å^–1^–1.29 Å^–1^ for IN16B and 0.46 Å^–1^–1.16 Å^–1^ for IRIS.

To describe internal protein dynamics detected by QENS the analytical model of the overdamped Brownian Oscillator given in eq 2 was applied.^37–39^ The factors *A*_0_(*q*) and *A*_*n*_(*q*) are introduced in (eqs 3 and 4), respectively. eq 5 defines the effective diffusion coefficient *D*_eff_ that provides information about molecular diffusive motions in BCA.

2 $$S{\left(q,\omega \right)}_{\mathrm{BO}}=A_{0}\left(q\right)\cdot \delta \left(\omega \right)+\sum_{n=1}^{N}A_{n}\left(q\right)\cdot \frac{1}{\pi }\cdot \frac{n\hbar D_{\mathrm{eff}}\left(q\right)/\left\langle x^{2}\right\rangle }{{\left(n\hbar D_{\mathrm{eff}}\left(q\right)/\left\langle x^{2}\right\rangle \right)}^{2}+{\left(\hbar \omega \right)}^{2}}$$

3 $$A_{0}\left(q\right)=e^{-\left\langle x^{2}\right\rangle q^{2}}\cdot \left(1-p\right)+p$$

4 $$A_{n}\left(q\right)=e^{-\left\langle x^{2}\right\rangle q^{2}}\cdot \left(1-p\right)\cdot \frac{{\left(q^{2}\left\langle x^{2}\right\rangle \right)}^{n}}{n!}$$

5 $$D_{\mathrm{eff}}\left(q\right)=\frac{D}{1+Dq^{2}\tau }$$

The assumed dynamics described by eq 5 accounts for molecular jump-diffusion with the diffusion coefficient *D* and the residence time τ. Pure diffusion-like behavior is retrieved for τ → 0. The two other parameters obtained from (eqs 3 and 4) are the mean square displacement (MSD) ⟨*x* ^2^⟩ and the apparently immobile fraction of hydrogen atoms of the protein *p*. The broadening of the individual spectra is described by ℏω.

For protein solutions, eq 2 needs to be convoluted by a narrow Lorentz function *L*(*q*, ω) that describes the effect of protein translational and rotational diffusion on the QENS spectra.^40–43^

6 $$S\left(q,\omega \right)=L\left(q,\omega \right)⊗S{\left(q,\omega \right)}_{\mathrm{BO}}$$

The line-widths Γ of the narrow Lorentzian (half-widths at half-maximum, HWHM) are given by

7 $$\Gamma \left(q\right)=D_{\mathrm{cm}}q^{2}$$

where *D*_cm_ is an effective diffusion coefficient that contains the contributions of both rotational and translational diffusion of the protein.^44–46^ In the accessible *q*-range of the used neutron spectrometers we assumed a *q* ^2^-dependence of Γ­(*q*) that is consistent with previous results as reported in the literature.^40–43,47^

The final theoretical model function then reads

8 $$S{\left(q,\omega \right)}_{\mathrm{theo}}=A_{0}\left(q\right)\cdot \frac{1}{\pi }\cdot \frac{\Gamma \left(q\right)}{\Gamma {\left(q\right)}^{2}+{\left(\hbar \omega \right)}^{2}}+\sum_{n=1}^{N}A_{n}\left(q\right)\cdot \frac{1}{\pi }\cdot \frac{n\hbar D_{\mathrm{eff}}/\left\langle x^{2}\right\rangle +\Gamma \left(q\right)}{{\left(n\hbar D_{\mathrm{eff}}/\left\langle x^{2}\right\rangle +\Gamma \left(q\right)\right)}^{2}+{\left(\hbar \omega \right)}^{2}}$$

eq 8 was convoluted with the measured instrumental resolution function and fitted to the experimental QENS data, with a python script based on jscatter,^48^ including a linear background in a global fit considering data of both instruments. The fits converged at *N*_sum_ = 50, with τ → 0. All fits were performed with the Levenberg–Marquardt (LM) algorithm. The uncertainties of the fitted parameters are reported as 1σ uncertainties and were determined from the square roots of the corresponding diagonal elements of the covariance matrix. Correlations between the fitted parameters were assessed using the corresponding correlation coefficients, which are provided in Table S4 of the SI.

## Results and Discussion

### ITC

The change in enthalpic values upon titration of PFBS and 4FBS with BCA in D_2_O and H_2_O at 298 and 308 K is shown in Figure 1. The representative raw exothermic thermograms and the corresponding integrated binding isotherms are shown in Figure S4A and B in the SI.

**Figure 1:**
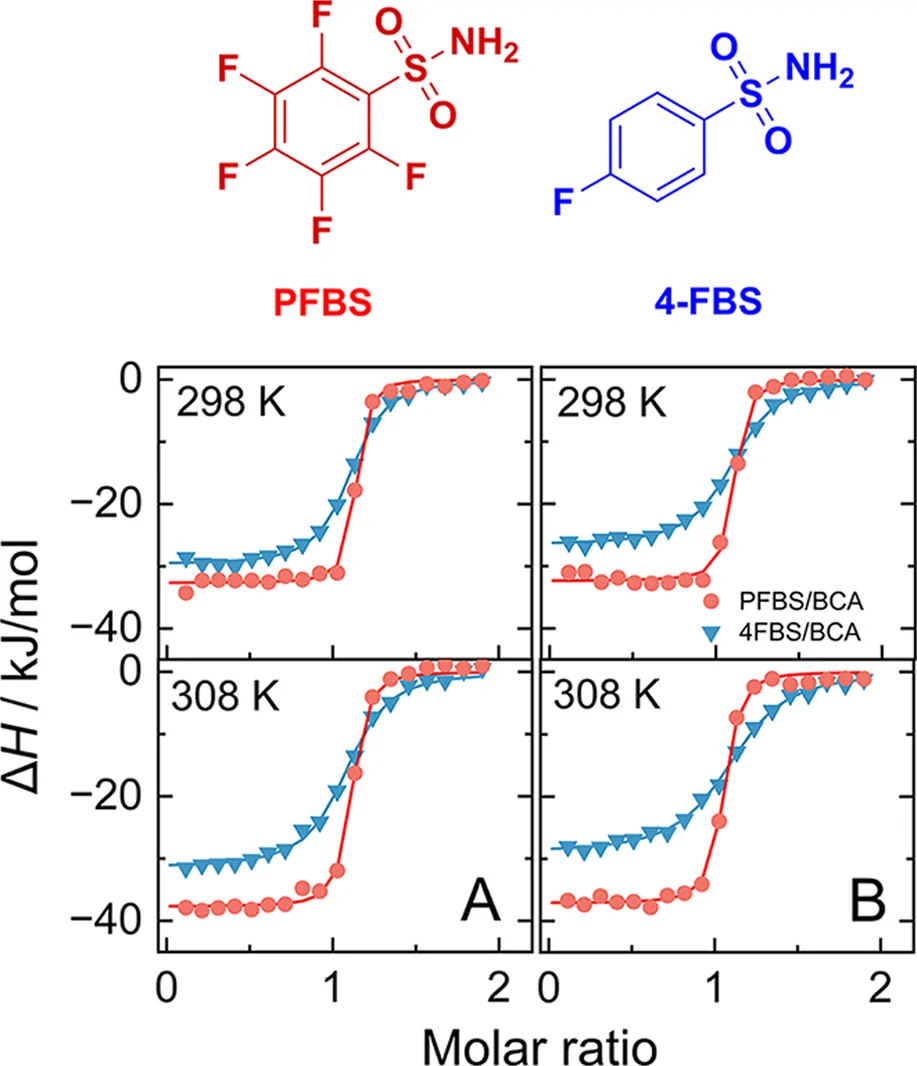
Chemical structures are shown on top of the plots. The integrated heat signal for the titration of PFBS (red) and 4FBS (blue) with BCA in (A) D_2_O and (B) H_2_O at various temperatures.

The sigmoidal enthalpic curves were fitted through a single site binding model for both ligands to obtain various thermodynamic parameters.^21,49^ As shown in Figure 2A, Δ*G* remains negative over the investigated temperature range, indicating that binding is spontaneous under all conditions examined. Notably, only modest changes in Δ*G* were observed with increasing temperature. On the other hand, increasing temperature results in more negative or favorable binding enthalpy but a less negative or reduced favorable entropy contribution. This case represents classical enthalpy–entropy compensation behavior observed for both ligand systems (see Figure 2B).^50,51^ Moreover, the more favorable or spontaneous binding of BCA/PFBS led to more negative Δ*G*, Δ*H*, and Δ*S* values compared to BCA/4FBS. However, for all cases, the binding is dominantly driven by enthalpy rather than entropy and the heat capacity values Δ*C*_*P*_ for exothermic binding was found to be negative (see Section S6 and Figure S5 in the SI). This has been attributed to the “non-classical hydrophobic effect”.^51^ However, the molecular basis of this hydrophobic effect in protein ligand binding is more complicated than this nonclassical description and not yet completely understood. Moreover, Snyder et al.^5^ showed that for the carbonic anhydrase-arylsulfonamide system, the negative Δ*C*_*P*_ cannot be just accounted for by simple nonpolar surface-area burial, and instead reflects the dehydration and reorganization of a specific, energetically frustrated water network confined within the hydrophobic wall of the active site.

**Figure 2:**
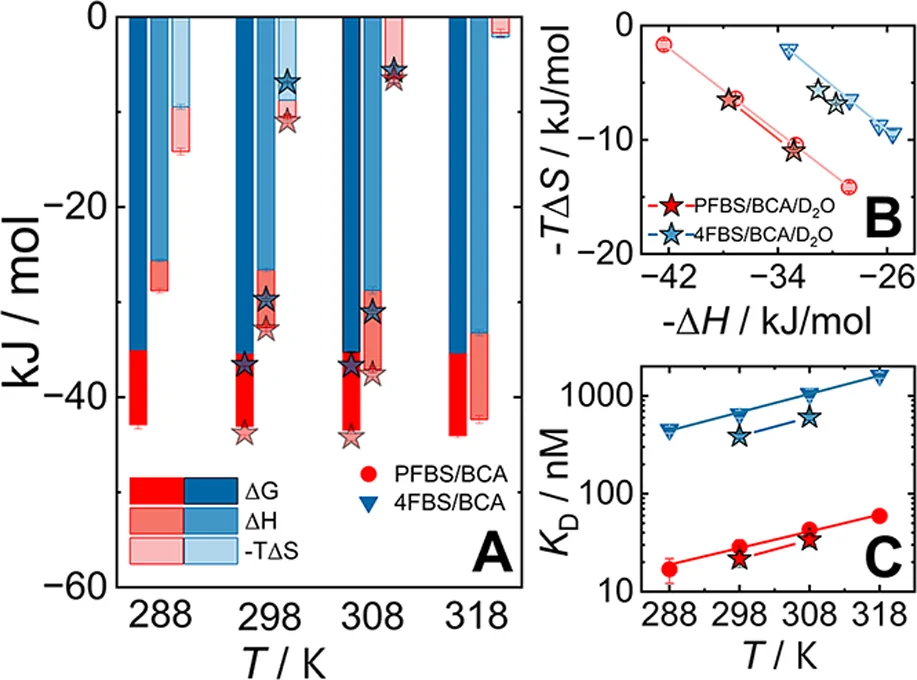
(A) Δ*G* (dark shade), Δ*H* (middle shade), – *T*Δ*S* (light shade) and (B) ITC data plotted on an enthalpy–entropy diagram. The *R* ^2^ values obtained from linear fits for PFBS/BCA and 4FBS/BCA were 0.999 and 0.998, respectively. (C) Dissociation constant *K*_D_ as a function of temperature. The lines are linear fits to the data. The star symbols represents measurements in D_2_O, whereas all other measurements were carried out in H_2_O. Note that the blue color corresponds to the 4FBS/BCA system while red color denotes the PFBS/BCA system.

The dissociation constant *K*_D_ increased with increasing temperature for both ligand systems (Figure 2C), indicating weaker binding at elevated temperatures. At comparable temperatures, PFBS bound substantially more strongly than 4FBS, with *K*_D_ values approximately 15–18 times lower. Comparison of the measurements in H_2_O and D_2_O revealed only modest solvent-isotope effects. The Δ*G* values differed by less than 5%, while Δ*H* and Δ*S* varied by approximately 1–15%. Nevertheless, the *K*_D_ values measured in D_2_O were consistently about 30% lower than those in H_2_O, indicating slightly stronger binding in the deuterated solvent. This is consistent with the stronger hydrogen-bonding network of D_2_O, which favors ligand association and slightly more exothermic binding enthalpies.^52^ Despite these quantitative differences, the characteristic enthalpy–entropy compensation and the temperature dependence of binding were essentially identical in both solvents, indicating that solvent isotope substitution does not alter the underlying binding mechanism. Furthermore, the thermodynamic parameters obtained in H_2_O are in good agreement with the values reported by Krishnamurthy et al.^53^

### QENS

In general, the QENS spectra of BCA solutions can be accurately described by assuming rigid-body diffusion of the protein and using the analytical model of the overdamped Brownian oscillator to describe its diffusive internal motion (see eq 8). The main features of the QENS spectra can be interpreted in terms of two characteristic contributions. The narrow Lorentzian component accounts for the global translational and rotational diffusion of the protein, whereas the broader quasielastic contribution describes the convolution between translational and rotational diffusion with localized internal protein dynamics on the ps-ns time scale. The widths and Q-dependence of these contributions provide information on the characteristic rates and spatial characteristics of the corresponding motions.^54^ Information on the MSD and immobile fraction of internal protein dynamics are contained within the respective amplitudes of both components. Representative QENS spectra of ligand-free BCA obtained with IN16B and IRIS, together with the corresponding theoretical fits, are shown in Figure 3. Global fitting of the QENS data recorded on both instruments enables the model parameters to be determined precisely. Representative QENS data including theoretical fits of ligand-free BCA, BCA+4FBS and BCA+PFBS are given in the SI in Figure S2 for 298 K and in Figure S3 for 308 K. The resulting fit parameters are shown in Figure 4 and are not correlated, the corresponding correlation coefficients are shown in Table S4 in the SI.

**Figure 3:**
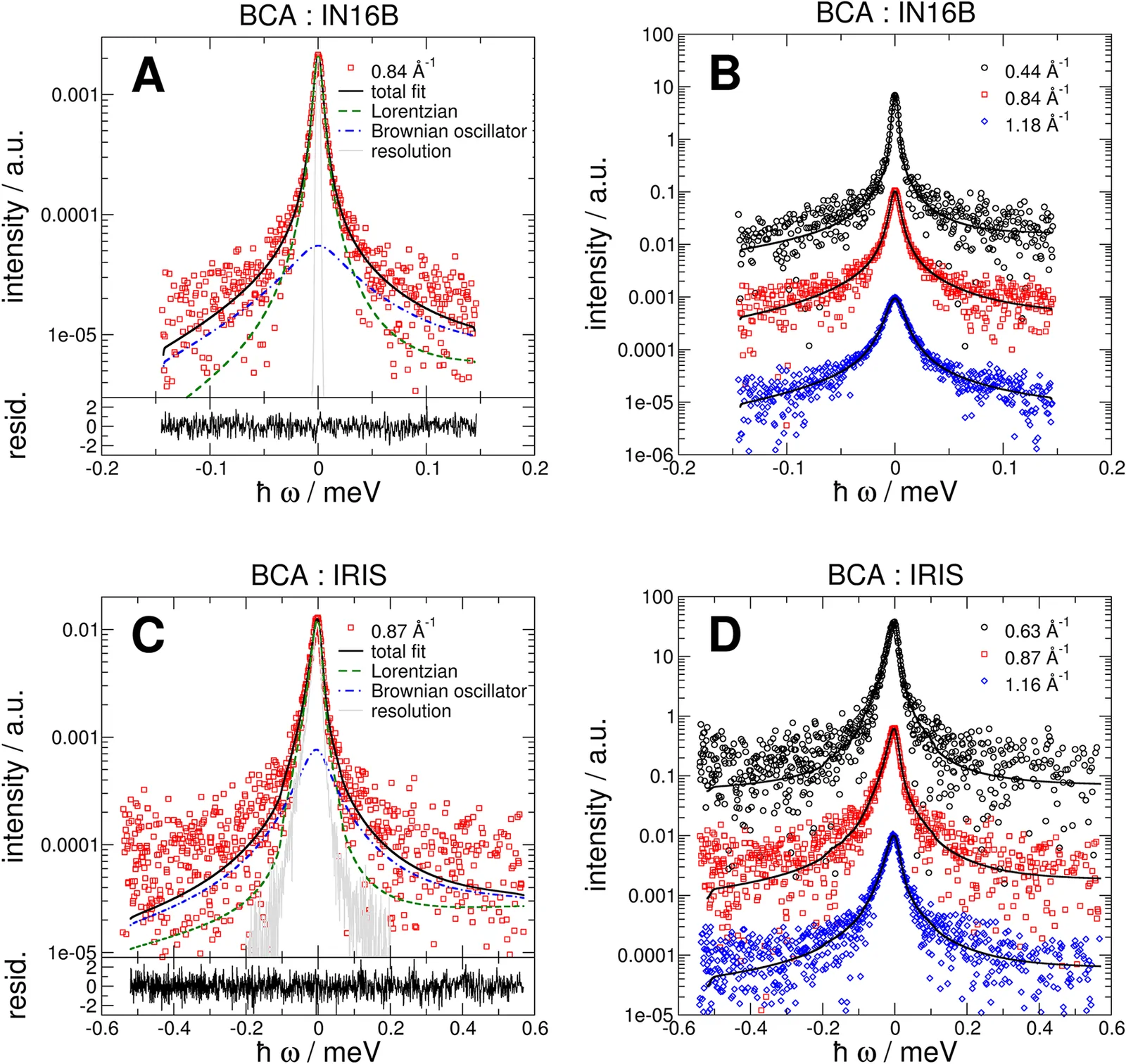
Experimental QENS data of ligand-free BCA at 298 K (symbols) including global fits using eq 8 (solid black lines). Selected QENS spectra measured on (A) IN16B using the BATS mode and (C) IRIS. The components of the theoretical model accounting for rigid-body diffusion (Lorentzian, green dashed line) and internal protein dynamics (Brownian oscillator, blue dashed-dotted line) as well as the instrumental resolution functions (solid gray lines) are shown in panels (A, C). Error-weighted residuals are given below panels (A) and (C) to illustrate the quality of the fit. Examples of experimental (B) IN16B and (D) IRIS spectra at selected *q*-values and global fits. Experimental data and fits shown in panels (B, D) are shifted consecutively by a factor of 50. For clarity only every second experimental data point is shown in all panels (A–D). All theoretical curves in panels (A-D) are convoluted with the instrumental resolution functions and linear background is added.

**Figure 4:**
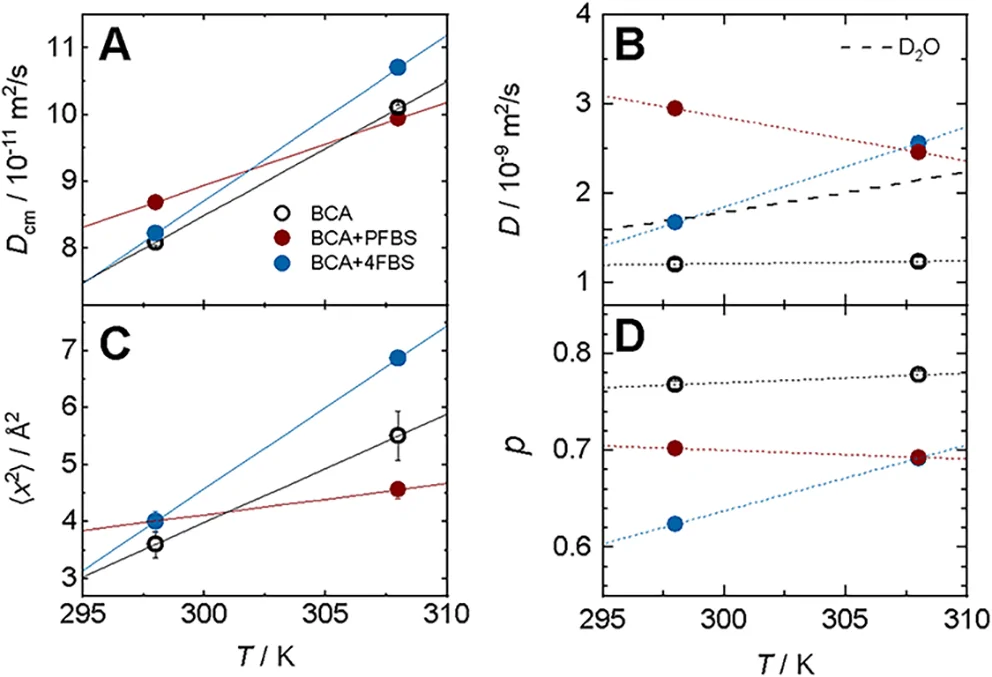
Global fit parameters: (A) *D*_cm_ the effective diffusion coefficient, (B) the effective internal diffusion coefficient *D* (see eq 5), (C) the mean square displacement ⟨x^2^⟩ as obtained from eqs 7, 8 and (D) immobile fraction of hydrogen atoms of the protein *p* (see eq 3) for BCA (black open circle), the complexes BCA+4FBS (blue bullet) and BCA+PFBS (red bullet). The dashed line represents literature values for D_2_O from eq 5 of ref 55.

First, we briefly discuss the effective diffusion coefficient *D*_cm_, whose values are plotted in Figure 4A and summarized in Table S2 in the SI. For free BCA and both complexes BCA+PFBS and BCA+4FBS *D*_cm_ increases from 298 to 308 K, most likely due to the decrease in solvent viscosity with increasing temperature (see Figure 4A). This is an expected result. For comparison, the Stokes–Einstein relation, using the temperature-dependent viscosity of D_2_O and *R*_h_ ≈ 2.1 nm for BCA, predicts an increase in the translational diffusion coefficient of approximately 30% between 298 and 308 K. Experimentally, *D*_cm_ increases from 8.09 × 10^–11^ to 10.1 × 10^–11^ m^2^ s^–1^, corresponding to an increase of 25%. The observed temperature dependence is therefore in good agreement with that expected from the Stokes–Einstein relation

Diffusion coefficients *D*, which characterize localized internal dynamics in BCA, are shown in Figure 4B and Table S2 in the SI. Ligand binding markedly enhances these dynamics. Upon PFBS binding, *D* increases by approximately a factor of 2 at both temperatures. A similar 2-fold increase is observed for the BCA+4FBS complex at 308 K, whereas the enhancement is less pronounced at 298 K. In contrast, the internal diffusion coefficient of ligand-free BCA remains essentially unchanged within experimental uncertainty between 298 and 308 K, demonstrating that the temperature dependence is induced by ligand binding rather than being an intrinsic property of the free enzyme.

To place these values into context, Figure 4B also includes the self-diffusion coefficient of D_2_O.^55^ Localized internal dynamics in ligand-free BCA are slower than the translational diffusion of the surrounding solvent. Upon ligand binding, however, this relationship changes substantially. For the BCA+4FBS complex at 298 K, the internal diffusion coefficient becomes comparable to that of the solvent, whereas for BCA+PFBS at both temperatures and for BCA+4FBS at 308 K, the localized internal dynamics are characterized by diffusion coefficients exceeding the self-diffusion coefficient of D_2_O. These findings demonstrate that ligand binding substantially redistributes the internal dynamical landscape of BCA.

The temperature dependence differs markedly between the two inhibitors. Whereas *D* decreases with increasing temperature for the BCA+PFBS complex, it increases for BCA+4FBS, indicating that the two ligands modulate the protein energy landscape in distinct ways. The stronger effect of PFBS may be related to its higher hydrophobicity (see Section S1 in the SI), which is expected to strengthen interactions with the hydrophobic region of the binding pocket.^53^ Such interactions could alter the balance between rigid and flexible regions of the protein and modify the surrounding hydration network, thereby changing the effective energy barriers governing localized dynamics. The opposite temperature trends observed for the two complexes suggest that ligand chemistry influences not only the magnitude of the internal dynamics but also their thermal response.

The apparently immobile fraction *p* is reduced upon ligand binding compared to free BCA, as given in Figure 4D and Table S2 in the SI. This shows that, due to ligand-binding, previously rigid residues in BCA have become more mobile in the complexes on the observed ps-ns time scale, as indicated by the increase in apparently mobile H atoms (1 – *p*). While BCA does not exhibit a significant temperature dependence of *p*, both BCA+PFBS and BCA+4FBS do. Interestingly, for BCA+PFBS, the decrease in *p* occurs in parallel with a reduction in *D* as a function of temperature. Compared to this, for BCA+4FBS, an increase in *p* with *T* is correlated with an increase in *D*. The reduction in *p* upon ligand binding indicates that a larger fraction of the protein contributes to motions within the time and length scales accessible to QENS. While this may reflect increased mobility of more strongly constrained residues, the QENS measurements do not provide residue-specific information and therefore do not allow the spatial location of these changes to be identified. The opposite temperature dependence of *D* and *p* observed for BCA+PFBS and BCA+4FBS indicates a ligand-specific response. Although the underlying mechanism remains to be elucidated, the correlated changes in both parameters suggest that they originate from the same physical process.

The MSD or ⟨x^2^⟩ is a measure of the flexibility of the protein. The results for all BCA samples measured with QENS are given in Figure 4C and Table S2 in the SI. At 298 K ligand-free BCA and both complexes BCA+4FBS and BCA+PFBS have a similar flexibility. As expected, we observe an increase of the MSD with temperature. Binding of 4FBS appears to result in a softer BCA+4FBS complex with a larger MSD value at 308 K compared to ligand-free BCA. Meanwhile, the BCA+PFBS complex is more rigid than free BCA at 308 K with a smaller MSD value.

Based on the measured ⟨x^2^⟩ the change in conformational entropy per residue Δ*S*_conf_/residue between the ligand-free and ligand-bound protein can be estimated by.^12,56,57^

9 $$\Delta S_{\mathrm{conf}}/\mathrm{residue}=3R\cdot \ln\left(\sqrt{\frac{\left\langle x_{\mathrm{BCA}+\mathrm{ligand}}^{2}\right\rangle }{\left\langle x_{\mathrm{BCA}}^{2}\right\rangle }}\right)$$

In our previous work,^12^ we multiplied Δ*S*_conf_/residue by the number of residues *N* to estimate Δ*S*_conf_ for the entire protein. This allowed for a direct comparison to the entropy determined by ITC.^12^ For streptavidin the apparently immobile fraction was found to be *p* = 0 on the ns time scale;^12^ therefore, this was an appropriate approach. For BCA, however, the apparently immobile fraction is nonzero (*p* > 0), indicating that not all residues contribute to the motions detected within the QENS time window. Therefore, simply scaling the conformational entropy contribution by the total number of residues *N* would overestimate the contribution from QENS-accessible motions. To account for this, we approximate the number of residues contributing to the conformational entropy by the number of apparently mobile residues, *N*(1 – *p*). Since the apparently immobile fraction *p* differs between the ligand-bound and ligand-free states, the weighting must be applied separately to the two states. The resulting weighted estimate of the conformational entropy change is therefore given by.

$$\Delta S_{\mathrm{conf}}=3R\left[N\left(1-p_{\mathrm{BCA}+\mathrm{ligand}}\right)\ln\left(\sqrt{\left\langle x_{\mathrm{BCA}+\mathrm{ligand}}^{2}\right\rangle }\right)-N\left(1-p_{\mathrm{BCA}}\right)\ln\left(\sqrt{\left\langle x_{\mathrm{BCA}}^{2}\right\rangle }\right)\right]$$

Using logarithm rules leads to

10 $$\Delta S_{\mathrm{conf}}=3R\cdot N\cdot \ln\left(\sqrt{\frac{\langle x_{\mathrm{BCA}+\mathrm{ligand}}^{2}\rangle^{(1-p_{\mathrm{BCA}+ligand})}}{\langle x_{\mathrm{BCA}}^{2}\rangle^{\left(1-p_{\mathrm{BCA}}\right)}}}\right)$$

in which the effective number of apparently mobile residues is used to estimate Δ*S*_conf_ for BCA and the BCA–ligand complex.

The resulting values are plotted in Figure 5A and listed in Table S3 (columns 3 and 4: Δ*S*_conf_ and −*T*Δ*S*_conf_) in the SI. Figure 5B compares the ligand-induced change in the conformational entropy of BCA with the corresponding change in the total binding entropy for PFBS and 4FBS at 298 and 308 K. In contrast to the study by Frederick et al. on calmodulin binding to different ligands, our results reveal pronounced differences between PFBS and 4FBS, suggesting that the two inhibitors induce distinct temperature-dependent changes in the conformational dynamics of the resulting protein–ligand complexes.

**Figure 5:**
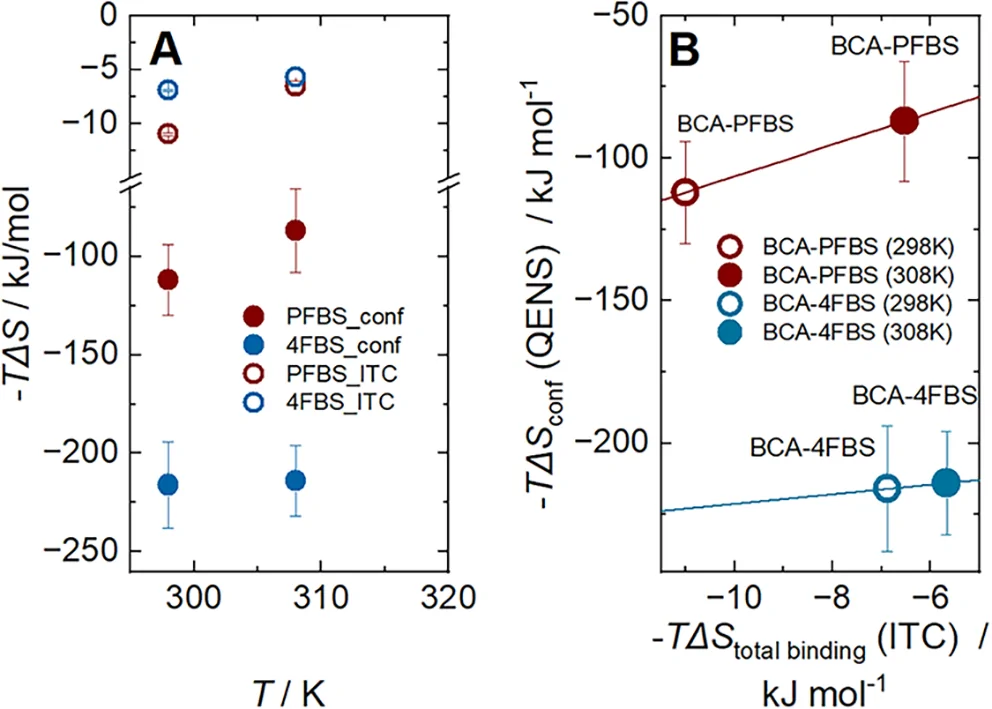
(A) −*T*Δ*S*_conf_ (bullet) and −*T*Δ*S*_ITC_ (open circle) for BCA binding to PFBS (red) and 4FBS (blue) in D_2_O as a function of temperature. (B) Correlation of the change in conformational entropy of BCA with the change in the total entropy of binding with PFBS and 4FBS at 298 and 308 K.

It should be noted that this approach to calculating Δ*S*_conf_ is a first-order approximation and neglects the contribution of slower motions that lie outside the time window of the neutron spectrometers used in this study. A study using neutron spin–echo spectroscopy provided evidence that slower protein motions might contribute to Δ*S*_conf_ ^58^ in maltose-binding protein but to a smaller extent. With regard to human CA, active-site loop motions were observed on the μs time scale. These motions are reduced in the inhibitor-bound state due to multiple hydrogen-bonds, as reported in Singh et al.^20^ Consequently, a protein-dependent scaling factor may be required to achieve a quantitative comparability between entropy changes derived from QENS and those obtained by calorimetric measurements.

### Integrating Thermodynamics and Protein Dynamics

ITC measurements in both D_2_O and H_2_O revealed only minor solvent isotope effects, justifying a direct comparison of the thermodynamic and dynamical data.

The results demonstrate that binding of PFBS and 4FBS to BCA is predominantly enthalpy driven, with only a modest favorable entropic contribution. For both ligands, the binding affinity decreases slightly with increasing temperature and is accompanied by a progressive redistribution of thermodynamic contributions: the favorable binding enthalpy becomes more negative, whereas the favorable entropy contribution −*T*Δ*S*_ITC_ decreases, reflecting classical enthalpy–entropy compensation. This compensation is substantially more pronounced for PFBS than for 4FBS (Figure 2A,B), possibly reflecting differences in the hydrophobicity and associated solvation behavior of the two ligands.

QENS complements this thermodynamic picture by revealing ligand-specific changes in protein dynamics on the ps–ns time scale. Increasing temperature affects both the internal diffusion coefficient *D* and the fraction of mobile hydrogen atoms *p* in a ligand-dependent manner (Figure 4B and D). Whereas binding of 4FBS results in a more flexible complex with a larger MSD at 308 K, PFBS produces a comparatively more rigid complex (Figure 4C). These contrasting responses show that the effect of ligand binding on the internal dynamics of BCA is ligand-specific and temperature-dependent, with PFBS and 4FBS producing distinct changes in the average conformational mobility of the protein.

To relate these dynamical changes to binding thermodynamics, we introduced a weighted estimate of the conformational entropy contribution, −*T*Δ*S*_conf_, that accounts for the fact that only a fraction of protein residues appears mobile within the time window accessible to QENS. Consistent with previous studies,^18,19^ both ligands increase the conformational entropy of BCA, indicating that complex formation broadens the ensemble of conformational states sampled by the protein. More importantly, the temperature dependence of −*T*Δ*S*_conf_ closely parallels that of the ITC-derived binding entropy, −*T*Δ*S*_ITC_ (Figure 5B). The agreement between these two independent observables, probing distinct time scales and length scales, indicates that fast protein motions are closely linked to the temperature dependence of the overall binding thermodynamics.

Together with the negative heat-capacity changes Δ*C*_*P*_, these observations are consistent with substantial reorganization of the protein–ligand–solvent interface accompanying ligand binding, whose thermodynamic consequences become increasingly pronounced with increasing temperature. Furthermore, TDFRS measurements showed that ligand binding displaces active-site water, making the complexes slightly less hydrophilic than ligand-free BCA.^14^ Within the experimental uncertainty of the TDFRS measurements, no significant difference in the hydrophilicity of the PFBS and 4FBS complexes was observed, despite their markedly different binding affinities. This similarity at the level of the complexes contrasts with the partitioning behavior of the free ligands. Krishnamurthy et al.^53^ reported octanol/phosphate buffer (pH 7.5) partition coefficients of 0.53 for 4FBS and 1.05 for PFBS, indicating a greater tendency of PFBS to partition into the organic phase under these conditions. Since these values were determined using phosphate buffer rather than pure water, they also reflect the ionization states of the inhibitors at pH 7.5. Thus, differences in the partitioning behavior of the free ligands do not translate directly into corresponding differences in the overall hydration properties of the protein–ligand complexes.

## Conclusion

By combining temperature-dependent ITC and QENS, we show that the thermodynamics of BCA inhibition cannot be understood from binding affinity or static protein structures alone. PFBS and 4FBS share the same benzenesulfonamide scaffold and differ in the degree of fluorination of the aromatic ring with one fluorine substituent in 4FBS and five in PFBS. Despite their similar overall binding modes, the two ligands induce distinct temperature-dependent changes in protein dynamics. The close correspondence between the temperature dependence of the QENS-derived estimate of conformational entropy and the ITC-derived binding entropy, together with the negative heat-capacity changes, indicates that enzyme-ligand recognition is governed by coupled changes in protein flexibility and reorganization of the protein–ligand-solvent interface.

Our results further show that changes in protein conformational dynamics do not occur in isolation but are closely linked to other entropic contributions accompanying ligand binding. Rather than arising from independent molecular components, the thermodynamics of binding reflects the coupled response of the protein–ligand-water system.

More broadly, these findings establish protein dynamics and hydration not as independent contributors, but as coupled aspects of a unified molecular response that governs temperature-dependent enzyme-ligand recognition.

## Supplementary Material



## Data Availability

The neutron scattering data are available from 10.5286/ISIS.E.RB2420502 for the IRIS data and from 10.5291/ILL-DATA.8-04-945 and 10.5291/ILL-DATA.EASY-1709 for the IN16B data. The ITC data of this study are available from Zenodo 10.5281/zenodo.21451114.

## Notes

Ligand log *P* values; concentrations used in the QENS experiments; determination of the *q*-range used for QENS analysis; QENS data and global fits; global QENS fit parameters and their correlation coefficients; raw data,tabulated thermodynamic parameters and Δ*C*_*P*_ values derived from the ITC experiments; and calculation of the ligand concentrations required to achieve near-complete protein binding (PDF)

## References

[1] Maren T. H. (1987). Carbonic
anhydrase: General perspective and advances in glaucoma research. Drug Dev. Res..

[2] Yang J., Koruza K., Fisher Z., Knecht W., Baltzer L. (2017). Improved molecular
recognition of Carbonic Anhydrase IX by polypeptide conjugation to
acetazolamide. Bioorg. Med. Chem..

[3] Fox J. M., Kang K., Sastry M., Sherman W., Sankaran B., Zwart P. H., Whitesides G. M. (2017). Water-Restructuring
Mutations Can
Reverse the Thermodynamic Signature of Ligand Binding to Human Carbonic
Anhydrase. Angew. Chem., Int. Ed..

[4] Fisher Z., Prada J. A. H., Tu C., Duda D., Yoshioka C., An H., Govindasamy L., Silverman D. N., McKenna R. (2005). Structural and kinetic
characterization of active-site histidine as a proton shuttle in catalysis
by human carbonic anhydrase II. Biochemistry.

[5] Snyder P. W., Mecinovic J., Moustakas D. T., Thomas S. W., Harder M., Mack E. T., Lockett M. R., Héroux A., Sherman W., Whitesides G. M. (2011). Mechanism of the hydrophobic effect
in the biomolecular recognition of arylsulfonamides by carbonic anhydrase. Proc. Natl. Acad. Sci. U.S.A..

[6] Mecinović J., Snyder P. W., Mirica K. A., Bai S., Mack E. T., Kwant R. L., Moustakas D. T., Héroux A., Whitesides G. M. (2011). Fluoroalkyl and Alkyl Chains Have
Similar Hydrophobicities
in Binding to the “Hydrophobic Wall” of Carbonic Anhydrase. J. Am. Chem. Soc..

[7] Kim J. K., Lim S. W., Jeong H. (2025). Fast product release
requires active-site water dynamics in carbonic anhydrase. Nat. Commun..

[8] Lindskog S. (1997). Structure
and mechanism of carbonic anhydrase. Pharmacol.
Ther..

[9] Frederick K. K., Marlow M. S., Valentine K. G., Wand A. J. (2007). Conformational entropy
in molecular recognition by proteins. Nature.

[10] Wienen-Schmidt B., Jonker H. R., Wulsdorf T., Gerber H. D., Saxena K., Kudlinzki D., Sreeramulu S., Parigi G., Luchinat C., Heine A., Schwalbe H., Klebe G. (2018). Paradoxically, Most
Flexible Ligand Binds Most Entropy-Favored: Intriguing Impact of Ligand
Flexibility and Solvation on Drug-Kinase Binding. J. Med. Chem..

[11] Verteramo M. L., Stenström O., Ignjatović M.
M., Caldararu O., Olsson M. A., Manzoni F., Leffler H., Oksanen E., Logan D. T., Nilsson U. J., Ryde U., Akke M. (2019). Interplay
between Conformational Entropy and Solvation Entropy in Protein-Ligand
Binding. J. Am. Chem. Soc..

[12] Sarter M., Niether D., Koenig B. W. (2020). Strong Adverse Contribution
of Conformational Dynamics to Streptavidin-Biotin Binding. J. Phys. Chem. B.

[13] Sarter M., Niether D., Wiegand S., Fitter J., Stadler A. M. (2022). Complementary
approaches to obtaining thermodynamic parameters from protein ligand
systems-challenges and opportunities. EPJ Web
Conf..

[14] Mohanakumar S., Lee N., Wiegand S. (2022). Complementary
Experimental Methods to Obtain Thermodynamic
Parameters of Protein Ligand Systems. Int. J.
Mol. Sci..

[15] Niether D., Sarter M., Koenig B. W. B., Fitter J., Stadler A. M. A., Wiegand S. (2020). Thermophoresis: The Case of streptavidin and biotin. Polymers.

[16] Gabel F., Masson P., Froment M. T., Doctor B. P., Saxena A., Silman I., Zaccai G., Weik M. (2009). Direct correlation
between molecular dynamics and enzymatic stability: A comparative
neutron scattering study of native human butyrylcholinesterase and
its “aged” soman conjugate. Biophys.
J..

[17] Trapp M., Trovaslet M., Nachon F., Koza M. M., van Eijck L., Hill F., Weik M., Masson P., Tehei M., Peters J. (2012). Energy landscapes of human acetylcholinesterase
and
its Huperzine A-inhibited counterpart. J. Phys.
Chem. B.

[18] Stöckmann H., Bronowska A., Syme N. R., Thompson G. S., Kalverda A. P., Warriner S. L., Homans S. W. (2008). Residual Ligand Entropy in the Binding
of p-Substituted Benzenesulfonamide Ligands to Bovine Carbonic Anhydrase
II. J. Am. Chem. Soc..

[19] Krishnamurthy V. M., Raman V. S., Mowery R. A., Hentz M., Baleja J. D., Shaw B. F., Kumar K. (2013). Ligand-Induced
Protein Mobility in
Complexes of Carbonic Anhydrase II and Benzenesulfonamides with Oligoglycine
Chains. PLoS One.

[20] Singh H., Das C. K., Vasa S. K., Grohe K., Schäfer L. V., Linser R. (2020). The Active Site of a Prototypical “Rigid”
Drug Target is Marked by Extensive Conformational Dynamics. Angew. Chem., Int. Ed..

[21] Krishnamurthy V. M., Kaufman G. K., Urbach A. R., Gitlin I., Gudiksen K. L., Weibel D. B., Whitesides G. M. (2008). Carbonic anhydrase as a model for
biophysical and physical-organic studies of proteins and protein-ligand
binding. Chem. Rev..

[22] Kasinath V., Sharp K. A., Wand A. J. (2013). Microscopic
insights into the NMR
relaxation-based protein conformational entropy meter. J. Am. Chem. Soc..

[23] Wand A. J. (2013). The dark
energy of proteins comes to light: conformational entropy and its
role in protein function revealed by NMR relaxation. Curr. Opin. Struct. Biol..

[24] Nyman P.-O., Lindskog S. (1964). Amino acid composition of various
forms of bovine and
human erythrocyte carbonic anhydrase. Biochim.
Biophys. Acta, Spec. Sect. Enzymol. Subj..

[25] Velazquez-Campoy, A. ; Leavitt, S. A. ; Freire, E. Characterization of protein-protein interactions by isothermal titration calorimetry. In Protein-Protein Interactions, Methods in Molecular Biology; Springer, 2004; Vol. 261, pp 35–54. 10.1385/1-59259-762-9:035 15064448

[26] Song H., Wilson D. L., Farquhar E. R., Lewis E. A., Emerson J. P. (2012). Revisiting
Zinc Coordination in Human Carbonic Anhydrase II. Inorg. Chem..

[27] Baranauskienė L., Petrikaite V., Matuliene J., Matulis D. (2009). Titration calorimetry
standards and the precision of isothermal titration calorimetry data. Int. J. Mol. Sci..

[28] Thomson, J. A. ; Ladbury, J. E. Isothermal Titration Calorimetry: A Tutorial. Biocalorimetry 2; Ladbury, J. E. ; Doyle, M. L. , Eds.; Wiley & Sons Ltd.: Chicester, 2004; pp 35–58.

[29] Brown A. (2009). Analysis of
Cooperativity by Isothermal Titration Calorimetry. Int. J. Mol. Sci..

[30] Appel M., Frick B., Magerl A. (2019). First results with the neutron backscattering
and TOF spectrometer option BATS on IN16B. Phys.
B: Condens. Matter.

[31] van
Eijck L., Gérard L., Frick B., Seydel T., Schober H. (2012). A case study for using neutron backscattering instruments
at reactors in inverted time-of-flight mode. Nucl. Instrum. Methods Phys. Res. A..

[32] Carlile C. J., Adams M. A. (1992). The design of the IRIS inelastic
neutron spectrometer
and improvements to its analysers. Phys. B:
Condens. Matter.

[33] Arnold O., Bilheux J., Borreguero J. (2014). Mantid - Data analysis
and visualization package for neutron scattering and *μ* SR experiments. Nucl. Instrum. Methods Phys.
Res. A..

[34] Project, M. Manipulation and Analysis Toolkit for Instrument Data. https://docs.mantidproject.org/v6.12.0/release/v6.12.0/index.html.

[35] Mantid IndirectILLEnergyTransfer algorithm 2026 https://docs.mantidproject.org/v6.7.0/algorithms/IndirectILLEnergyTransfer-v1.html. accessed July 1.

[36] Sarter M., Stewart J. R., Nilsen G. J., Devonport M., Nemkovski K. (2024). Data Analysis of Dynamics in Protein
Solutions Using
Quasi-Elastic Neutron Scattering-Important Insights from Polarized
Neutrons. J. Am. Chem. Soc..

[37] Volino F., Perrin J.-C., Lyonnard S. (2006). Gaussian model for localized translational
motion: application to incoherent neutron scattering. J. Phys. Chem. B.

[38] Kneller G. R. (2005). Quasielastic
neutron scattering and relaxation processes in proteins: analytical
and simulation-based models. Phys. Chem. Chem.
Phys..

[39] Stadler A.
M., Garvey C. J., Embs J. P., Koza M. M., Unruh T., Artmann G., Zaccai G. (2014). Picosecond dynamics in haemoglobin
from different species: A quasielastic neutron scattering study. Biochim. Biophys. Acta, Gen. Subj..

[40] Ameseder F., Radulescu A., Khaneft M., Lohstroh W., Stadler A. M. (2018). Homogeneous
and heterogeneous dynamics in native and denatured bovine serum albumin. Phys. Chem. Chem. Phys..

[41] Monkenbusch M., Stadler A., Biehl R., Ollivier J., Zamponi M., Richter D. (2015). Fast internal dynamics in alcohol dehydrogenase. J. Chem. Phys..

[42] Grimaldo M., Roosen-Runge F., Zhang F., Schreiber F., Seydel T. (2019). Dynamics of proteins
in solution. Q. Rev. Biophys..

[43] Roosen-Runge F., Hennig M., Zhang F., Jacobs R. M. J., Sztucki M., Schober H., Seydel T., Schreiber F. (2011). Protein self-diffusion
in crowded solutions. Proc. Natl. Acad. Sci.
U.S.A..

[44] Stadler A., Digel I., Artmann G., Embs J., Zaccai G., Büldt G. (2008). Hemoglobin dynamics in red blood cells: Correlation
to body temperature. Biophys. J..

[45] Pérez J., Zanotti J.-M., Durand D. (1999). Evolution of the Internal
Dynamics
of Two Globular Proteins from Dry Powder to Solution. Biophys. J..

[46] Biehl R., Monkenbusch M., Richter D. (2011). Exploring internal
protein dynamics
by neutron spin echo spectroscopy. Soft Matter.

[47] Shou K., Sarter M., de Souza N. R., de Campo L., Whitten A. E., Kuchel P. W., Garvey C. J., Stadler A. M. (2020). Effect of red blood
cell shape changes on haemoglobin interactions and dynamics: a neutron
scattering study. R. Soc. Open Sci..

[48] Biehl R. (2019). Jscatter,
a program for evaluation and analysis of experimental data. PLoS One.

[49] Rogez-Florent T., Duhamel L., Goossens L., Six P., Drucbert A.-S., Depreux P., Danzé P.-M., Landy D., Goossens J.-F., Foulon C. (2014). Label-free characterization
of carbonic anhydrase–novel
inhibitor interactions using surface plasmon resonance, isothermal
titration calorimetry and fluorescence-based thermal shift assays. J. Mol. Recognit..

[50] Annual Review of Biophysics, Vol 42; Dill, K. A. , Ed.; Annual Review of Biophysics, 2013; Vol. 42, pp 121–142.

[51] Paul B. K. (2022). Classical
vs. nonclassical hydrophobic interactions underlying various interaction
processes: Application of isothermal titration calorimetry. Chem. Phys. Impact.

[52] Giubertoni G., Bonn M., Woutersen S. (2023). D2O as an
Imperfect Replacement for
H2O: Problem or Opportunity for Protein Research?. J. Phys. Chem. B.

[53] Krishnamurthy V. M., Bohall B. R., Kim C.-Y., Moustakas D. T., Christianson D. W., Whitesides G. M. (2007). Thermodynamic parameters for the
association of fluorinated benzenesulfonamides with bovine carbonic
anhydrase II. Chem. - Asian J..

[54] Gabel F., Bicout D., Lehnert U., Tehei M., Weik M., Zaccai G. (2002). Protein dynamics studied by neutron scattering. Q. Rev. Biophys..

[55] Edura Y., Morishima N. (2005). Cold and thermal neutron scattering
in liquid water–II:
Scattering laws and group constants for H2O and D2O. Nucl. Instrum. Methods Phys. Res., Sect. A.

[56] Fitter J. (2003). A Measure
of Conformational Entropy Change during Thermal Protein Unfolding
Using Neutron Spectroscopy. Biophys. J..

[57] Receveur V., Calmettes P., Smith J. C., Desmadril M., Coddens G., Durand D. (1997). Picosecond
dynamical changes on denaturation
of yeast phosphoglycerate kinase revealed by quasielastic neutron
scattering. Proteins.

[58] Shukla S., Stingaciu L.-R., Stanley C., Agarwal P., Cuneo M., Stadler A. M., Myles D. (2025). Dynamical Signatures of Thermotoga
maritima Maltose-Binding Proteins Affected by Ligand Binding. ACS Omega.

