# Supplementary material for: Temperature-Dependent Enzyme-Ligand Recognition Emerges from the Coupling of Protein Dynamics and Hydration
Source: ACS Omega. 2026 Oct 21;11(43):67657–65. doi: 10.1021/acsomega.6c09199 (PMC13737023; doi:10.1021/acsomega.6c09199)
Supplement: Supplementary file 1 [file ao6c09199_si_001.pdf]

Supporting information: Temperature-dependent enzyme–ligand recognition emerges from the coupling of protein dynamics and hydration

Binny A. Rudani,[*,†,#] Mona Sarter,[*,‡,#] Tilo Seydel,[¶] Simone Wiegand,[*,†,§] and Andreas M. Stadler[*,‖,⊥]

[†] *Institute of Biological Information Processing IBI-4 IBI-4:Biomacromolecular Systems and Processes, Forschungszentrum Jülich GmbH, 52425 Jülich, Germany*

[‡] *ISIS Neutron and Muon Facility, STFC Rutherford Appleton Laboratory, Didcot OX11 0QX, U.K.*

[¶] *Institut Laue-Langevin, 71 Avenue des Martyrs, CS 20156, 38042 Grenoble Cedex 9, France*

[§] *Department für Chemie - Institut für Licht und Materialien, Universität zu Köln, 50939 Cologne, Germany*

[‖] *Jülich Centre for Neutron Science JCNS-1: Neutron Scattering and Soft Matter, Forschungszentrum Jülich GmbH, 52425 Jülich, Germany*

[⊥] *Institute of Physical Chemistry, RWTH Aachen University, Landoltweg 2, 52056 Aachen, Germany*

[#] *B.A.R and M.S. contributed equally*

E-mail: b.rudani@fz-juelich.de; mona.sarter@stfc.ac.uk; s.wiegand@fz-juelich.de; a.stadler@fz-juelich.de

Contents

S1 $\log P$ **values of ligands** **S2**

S2 Corresponding concentrations for QENS **S2**

S3 Determing the q**-range for QENS** **S3**

S4 QENS data and global fits **S4**

S5 Parameters determined in the QENS experiments **S6**

S6 Raw data and thermodymanic parameters calculated from ITC experiment. **S7**

S7 Calculation of the Ligand Concentration for Complete Protein Binding **S11**

S1 $\log P$ values of ligands

In our study, we used pentafluorobenzenesulfonamide (PFBS) and 4-fluorobenzenesulfonamide (4FBS). $\log P$ values were calculated using the Calculator Plugins in Marvin 24.1.2 (ChemAxon) [1], based on the method of Vishwanadhan *et al.* [2]. Lower (or more negative) $\log P$ values indicate greater hydrophilicity [3]. The calculated $\log P$ values were 1.38 for PFBS and 0.82 for 4FBS, suggesting 4FBS is more hydrophilic compared to PFBS.

Krishnamurthy *et al.* [4] experimentally determined octanol/phosphate buffer (pH 7.5) partition coefficients of 1.05 for PFBS and 0.53 for 4FBS. These measurements likewise indicate a greater preference of PFBS for the organic phase. However, because phosphate buffer at pH 7.5 rather than pure water was used as the aqueous phase, these experimental values also depend on the ionization states of the inhibitors and therefore cannot be directly compared with the calculated standard $\log P$ values. Nevertheless, both approaches consistently indicate that PFBS has a greater tendency to partition into the organic phase than 4FBS.

S2 Corresponding concentrations for QENS

Table S1 lists the samples used for the QENS measurements and their corresponding concentrations. For both systems, the ligand concentration was sufficiently high that the fraction of free protein was below 1×10^{-5} for PFBS and 1.6×10^{-4} for 4FBS (see Section S7).

Table S1: Samples used for the QENS measurements and their corresponding concentrations.

Sample	Molar mass / $\mathrm{g\,mol^{-1}}$	[BCA] and [Ligand] / $\mathrm{mg\,mL^{-1}}$	[BCA] and [Ligand] / $\mathrm{mmol\,L^{-1}}$
BCA	29844	50	1.68
Free PFBS	247.14	0.63	2.55
BCA + PFBS	29844 / 247.14	50 / 1.05	1.68 / 4.25
Free 4FBS	175.18	0.72	4.11
BCA + 4FBS	29844 / 175.18	50 / 0.74	1.68 / 4.22

S3 Determing the q-range for QENS

To determine the q-range over which the data can be analyzed using the incoherent scattering approximation, the ratio of the integrated buffer signal to the integrated protein-buffer signal was plotted as a function of q. For reliable analysis, this ratio must remain below unity to ensure that the scattering is not dominated by coherent contributions [5]. The corresponding plot is shown in Figure S1. Based on this criterion, the usable q-range is 0.46 Å^{-1} to 1.16 Å^{-1} for IRIS and 0.44 Å^{-1} to 1.29 Å^{-1} for IN16B. This choice also avoids the pronounced increase in the ratio at higher q values, where coherent scattering from the hydration shell is no longer negligible.

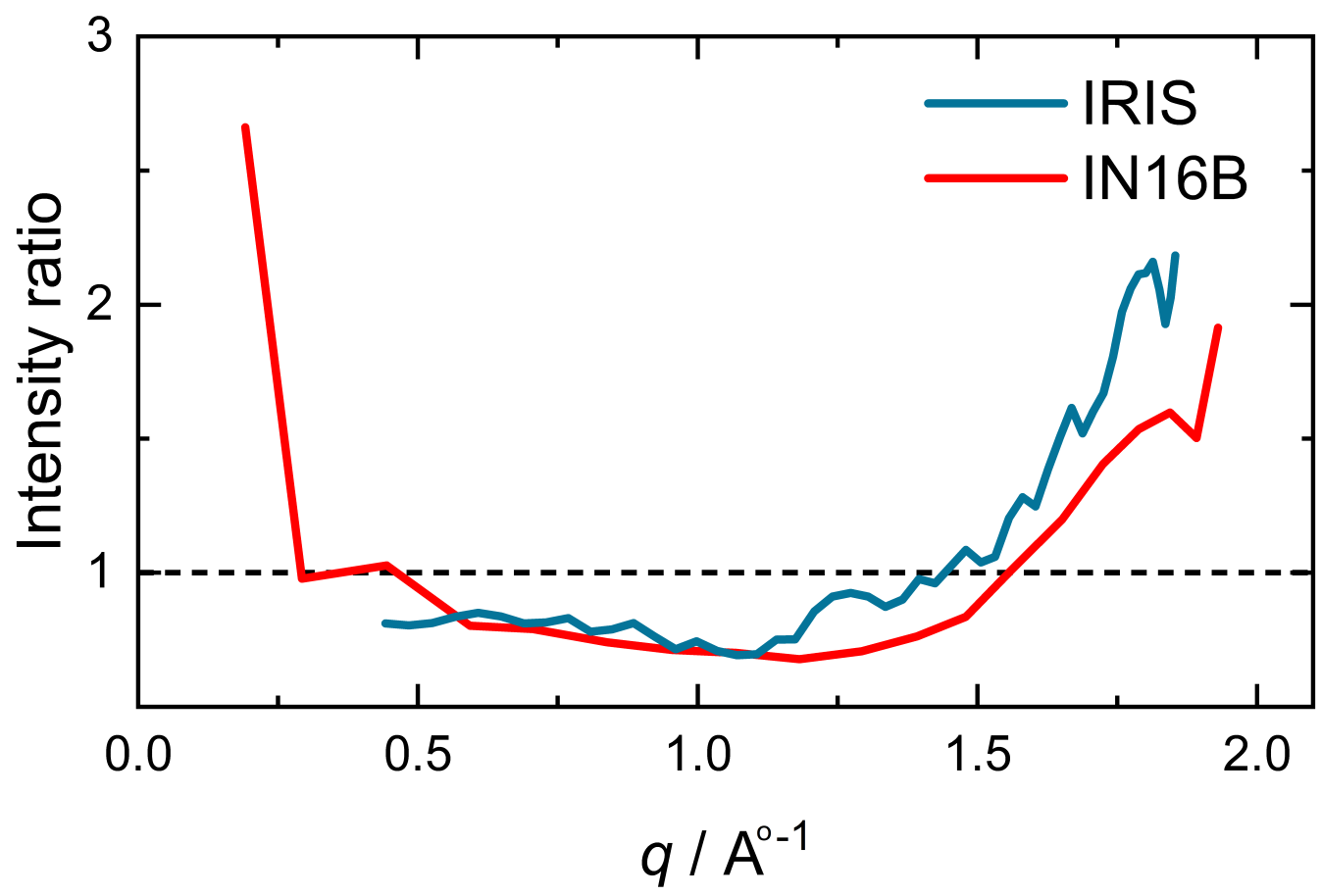

Figure S1: Ratio of the integrated buffer to protein signal used to determine the q-range. The ratio had to be below unity.

S4 QENS data and global fits

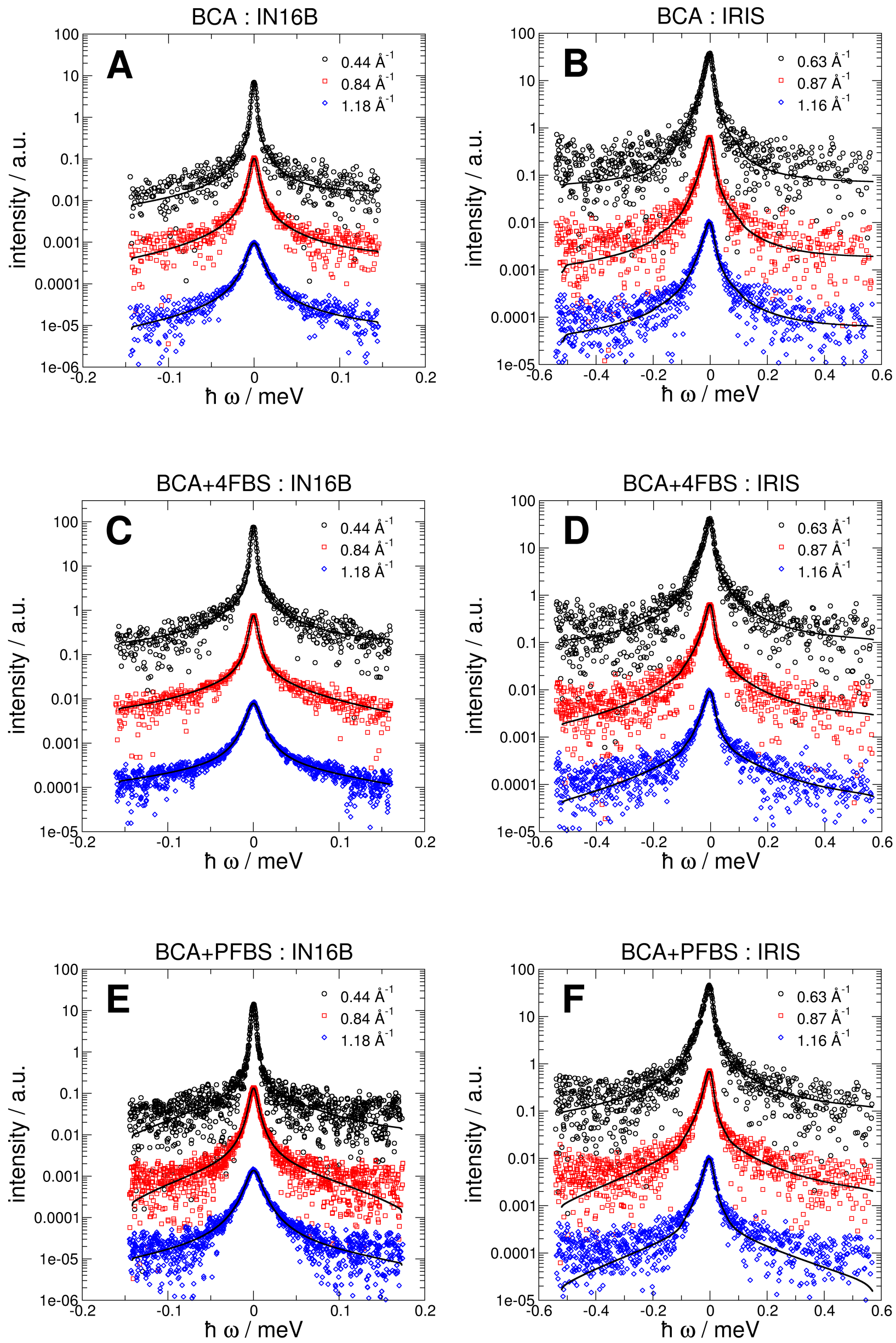

Figure S2: Experimental QENS data of (A,B) ligand-free BCA, (C,D) BCA+4FBS and (E,F) BCA+PFBS at 298 K at selected q-values (symbols) including global fits using Equation 8 (solid lines). (A,C,E) Selected QENS spectra measured on IN16B using the BATS mode including fits, (B,D,F) examples of experimental IRIS data at selected q-values and fits. For clarity only every second experimental data point is shown in all panels and experimental data and fits were shifted by factors of 250, 50, 1, respectively. Solid lines in the panels represent the results of the global fit approach to QENS data of both instruments.

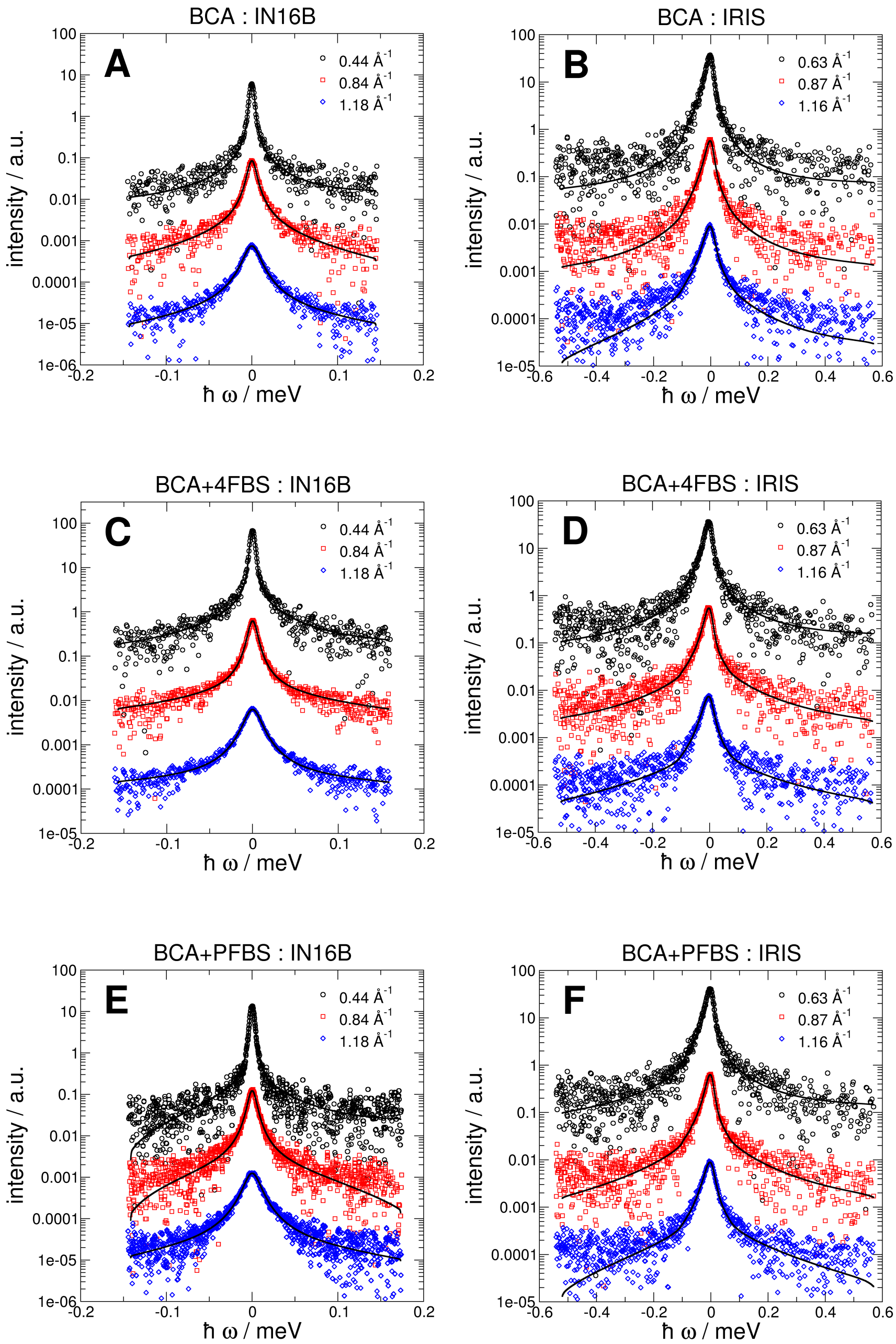

Figure S3: Experimental QENS data of (A,B) ligand-free BCA, (C,D) BCA+4FBS and (E,F) BCA+PFBS at 308 K at selected q-values (symbols) including global fits using Equation 8 (solid lines). (A,C,E) Selected QENS spectra measured on IN16B using the BATS mode including fits, (B,D,F) examples of experimental IRIS data at selected q-values and fits. For clarity only every second experimental data point is shown in all panels and experimental data and fits were shifted by factors 250, 50, 1 respectively. Solid lines in the panels represent the results of the global fit approach to QENS data of both instruments.

S5 Parameters determined in the QENS experiments

Table S2 lists the global fit parameters: D_{cm} the effective diffusion coefficient, the diffusion coefficient D describing the internal dynamics of the protein (see Equation 5 in main manuscript), the immobile fraction of hydrogen atoms of the protein p (see Equation 4 main manuscript) and the mean square displacement $\langle x^2 \rangle$ as obtained from Equations 3 and 4 in the main manuscript.

Table S2: Global fit parameters: effective diffusion coefficient D_{cm}, diffusion coefficient D, immobile fraction of hydrogen atoms of the protein p, and mean square displacement $\langle x^2 \rangle$.

Sample	T / K	D_{cm} / 10^{-11} $\mathrm{m^2\,s^{-1}}$	D / 10^{-11} $\mathrm{m^2\,s^{-1}}$	p	$\langle x^2 \rangle$ / Å^2
BCA	298	8.09 ± 0.04	120.9 ± 4.6	0.768 ± 0.004	3.60 ± 0.23
BCA	308	10.10 ± 0.05	123.9 ± 4.7	0.778 ± 0.004	5.51 ± 0.43
BCA + PFBS	298	8.69 ± 0.02	294.7 ± 6.1	0.702 ± 0.003	4.01 ± 0.12
BCA + PFBS	308	9.94 ± 0.03	246.1 ± 4.6	0.693 ± 0.003	4.56 ± 0.15
BCA + 4FBS	298	8.22 ± 0.08	167.4 ± 4.1	0.624 ± 0.004	4.00 ± 0.18
BCA + 4FBS	308	10.70 ± 0.08	256.6 ± 8.0	0.692 ± 0.005	6.87 ± 0.11

Table S3 lists $-T\Delta S_{\mathrm{conf}}$ and $-T\Delta S_{\mathrm{ITC}}$ for BCA binding to PFBS and 4FBS in D_2O which have been calculation according to Equation 10 in the main manuscript.

Table S3: $-T\Delta S_{\mathrm{conf}}$ and $-T\Delta S_{\mathrm{ITC}}$ for BCA binding to PFBS and 4FBS in D_2O. For the calculation of $-T\Delta S_{\mathrm{conf}}$, Equation 10 was used with a protein model comprising 259 amino acid residues. Additional details are provided in the text.

Sample	T / K	ΔS_{conf} / $\mathrm{J\,mol^{-1}\,K^{-1}}$	$-T\Delta S_{\mathrm{conf}}$ / $\mathrm{kJ\,mol^{-1}}$	ΔS_{ITC} / $\mathrm{J\,mol^{-1}\,K^{-1}}$	$-T\Delta S_{\mathrm{ITC}}$ / $\mathrm{kJ\,mol^{-1}}$
BCA + PFBS	298	377 ± 59	-112 ± 18	38 ± 0.5	-11.0 ± 0.14
BCA + PFBS	308	281 ± 69	-87 ± 21	21 ± 1.6	-6.53 ± 0.52
BCA + 4FBS	298	724 ± 73	-216 ± 22	23 ± 0.3	-6.88 ± 0.09
BCA + 4FBS	308	694 ± 59	-214 ± 18	18 ± 2	-5.67 ± 0.62

Table S4 shows the correlation coefficients obtained for the parameters shown in Figure 4 in the main manuscript. The results are all ≈ 0 and therefore indicate that the different parameters are not correlated.

Table S4: Correlation coefficients obtained from the covariance matrix for all parameters shown in Figure 4 in the main manuscript.

Sample	T/K	$\rho_{\langle x^2\rangle p}$	$\rho_{\langle x^2\rangle D_{\mathrm{CM}}}$	$\rho_{\langle x^2\rangle D}$	ρ_{pD}	$\rho_{pD_{\mathrm{CM}}}$	$\rho_{DD_{\mathrm{CM}}}$
BCA	298	$4.6 \cdot 10^{-5}$	$4.0 \cdot 10^{-5}$	$4.4 \cdot 10^{-5}$	$1.6 \cdot 10^{-4}$	$1.8 \cdot 10^{-4}$	$1.6 \cdot 10^{-4}$
BCA	308	$5.0 \cdot 10^{-5}$	$2.3 \cdot 10^{-4}$	$6.5 \cdot 10^{-5}$	$2.3 \cdot 10^{-4}$	$2.4 \cdot 10^{-4}$	$2.3 \cdot 10^{-4}$
BCA + PFBS	298	$-2.1 \cdot 10^{-4}$	$-2.0 \cdot 10^{-4}$	$-2.3 \cdot 10^{-4}$	$7.1 \cdot 10^{-4}$	$1.2 \cdot 10^{-3}$	$8.7 \cdot 10^{-4}$
BCA + PFBS	308	$-2.7 \cdot 10^{-4}$	$-2.2 \cdot 10^{-4}$	$-2.6 \cdot 10^{-4}$	$6.8 \cdot 10^{-4}$	$1.1 \cdot 10^{-3}$	$7.8 \cdot 10^{-4}$
BCA + 4FBS	298	$2.6 \cdot 10^{-5}$	$-1.5 \cdot 10^{-7}$	$6.5 \cdot 10^{-5}$	$1.6 \cdot 10^{-4}$	$1.6 \cdot 10^{-4}$	$1.6 \cdot 10^{-4}$
BCA + 4FBS	308	$7.5 \cdot 10^{-5}$	$3.1 \cdot 10^{-5}$	$1.2 \cdot 10^{-4}$	$2.7 \cdot 10^{-4}$	$2.5 \cdot 10^{-4}$	$2.4 \cdot 10^{-4}$

S6 Raw data and thermodymanic parameters calculated from ITC experiment.

Figure S4 shows the raw ITC thermograms and the corresponding integrated heat signals for PFBS/BCA and 4FBS/BCA in D_2O and H_2O, respectively. Note that the DP peak height reflects the total heat released or absorbed during each injection and therefore depends on the amount of material delivered. Consequently, the 4FBS/BCA signal is larger because the ligand and BCA concentrations were approximately twofold higher than those used in the PFBS/BCA experiment. Despite the lower concentrations employed for PFBS/BCA, titration of PFBS into BCA produced larger binding enthalpies than 4FBS at all temperatures.

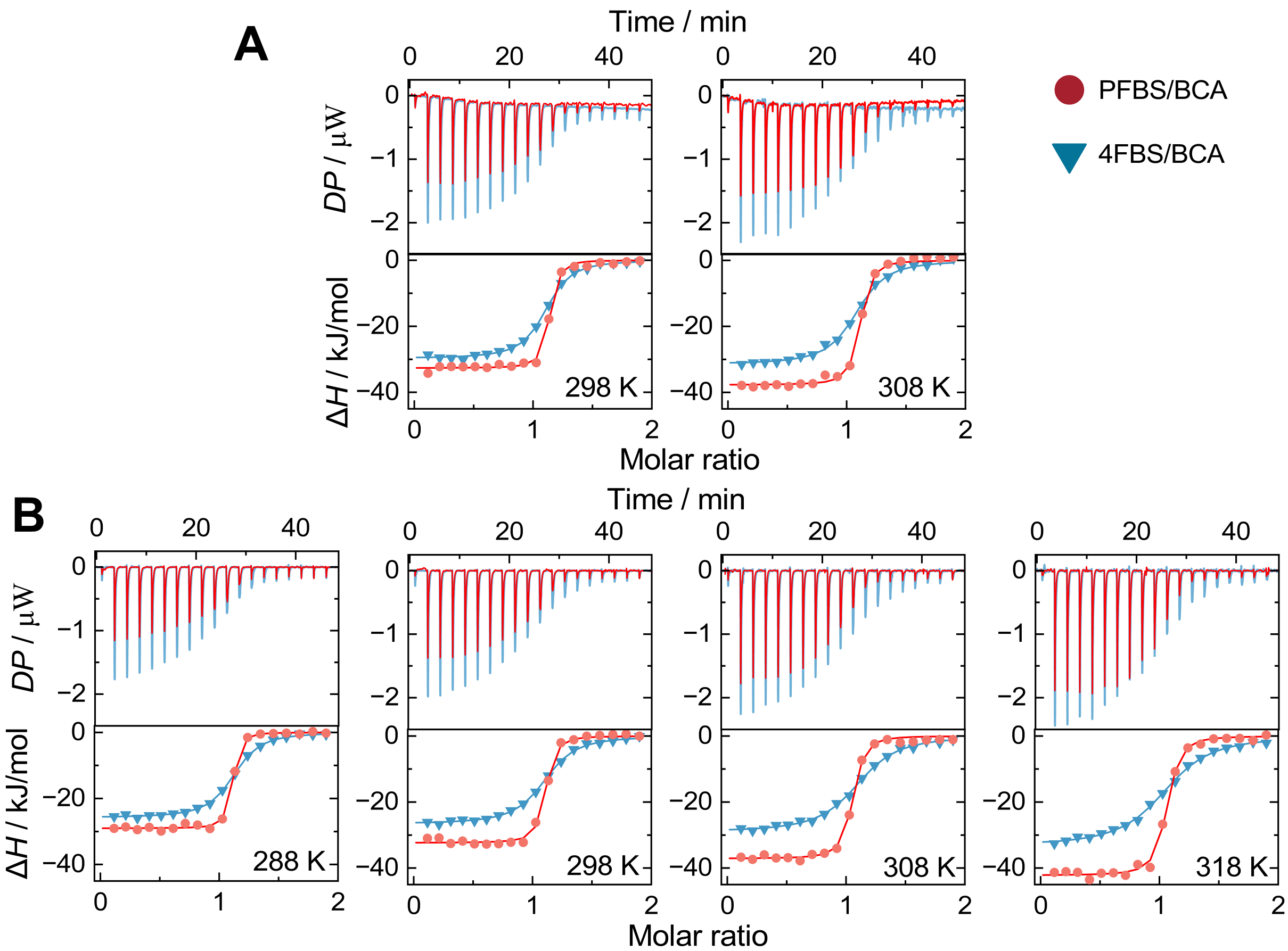

Figure S4: Raw data (top) and the integrated heat signal for the titration of PFBS (red) and 4FBS (blue) with BCA in (A) D_2O and (B) H_2O at various temperatures.

Table S5 and S6 summarize the thermodynamic parameters for 4FBS/BCA and PFBS/BCA obtained from ITC measurements in D_2O and H_2O, respectively. All the data were analyzed using the one-site binding model. The listed values are plotted in the main manuscript.

Table S5: Thermodynamic parameters of PFBS/BCA and 4FBS/BCA in D_2O determined by ITC using the one-site binding model. N was nearly 1.07 ± 0.002 indicating that the molecular binding ratio between BCA and inhibitors was about 1:1.

Sample	T / K	K_D / nM	ΔH / kJ/mol	ΔG / kJ/mol	$-T\Delta S$ / kJ/mol
PFBS/BCA	298	21.6 ± 3.9	-32.85 ± 0.19	-43.8 ± 0.14	-11.0 ± 0.14
PFBS/BCA	308	33.7 ± 5.2	-37.6 ± 0.24	-44.2 ± 0.35	-6.53 ± 0.52
4FBS/BCA	298	387 ± 22	-29.75 ± 0.14	-36.6 ± 0.14	-6.88 ± 0.09
4FBS/BCA	308	606 ± 42	-31.05 ± 0.20	-36.7 ± 0	-5.67 ± 0.62

Table S6: Thermodynamic parameters of PFBS/BCA and 4FBS/BCA determined by ITC in H_2O using the one-site binding model. N was nearly 1.07 ± 0.002, indicating that the molecular binding ratio between BCA and inhibitors was about 1:1

Sample	T / K	K_D / nM	ΔH / kJ/mol	ΔG / kJ/mol	$-T\Delta S$ / kJ/mol
PFBS/BCA	288	16.95 ± 4.81	-28.8 ± 0.24	-42.9 ± 0.42	-14.15 ± 0.35
PFBS/BCA	298	28.5 ± 5.49	-32.7 ± 0.35	-43.1 ± 0.14	-10.45 ± 0.21
PFBS/BCA	308	42.95 ± 5.58	-37.1 ± 0.32	-43.5 ± 0	-6.41 ± 0.021
PFBS/BCA	318	59.3 ± 7.78	-42.35 ± 0.42	-44.05 ± 0.21	-1.67 ± 0.38
4FBS/BCA	288	451 ± 35	-25.6 ± 0.16	-35 ± 0	-9.44 ± 0.27
4FBS/BCA	298	651 ± 57.6	-26.6 ± 0.22	-35.35 ± 0.07	-8.74 ± 0.014
4FBS/BCA	308	1050 ± 120.35	-28.75 ± 0.37	-35.25 ± 0.07	-6.51 ± 0.43
4FBS/BCA	318	1625 ± 113.5	-33.2 ± 0.31	-35.3 ± 0.14	-2.10 ± 0.042

In addition to the thermodynamic parameters discussed above, the change in heat capacity upon binding, ΔC_P, was determined [6, 7, 8, 9]. Values of ΔC_P were obtained from the temperature dependence of the binding enthalpy, ΔH, using the ITC data for both complexes in D_2O and H_2O (Tables S5 and S6). The resulting ΔC_P values are summarized in Figure S5 and were calculated according to equations:

$$\Delta C_P(D_2O) = \frac{\Delta H(308\,K) - \Delta H(298\,K)}{308 - 298}, \tag{S1}$$

$$\Delta C_P(H_2O) = \left(\frac{\partial \Delta H}{\partial T}\right)_p \tag{S2}$$

The thermodynamic signatures of PFBS and 4FBS binding to BCA, characterized by large favorable enthalpies ($\Delta H = -32.85$ and -29.75 kJ mol^{-1} at 298 K, respectively), smaller but favorable entropic contributions ($-T\Delta S = -11.0$ and -6.88 kJ mol^{-1}), and negative heat capacity changes ($\Delta C_P < 0$), are consistent with the non-classical hydrophobic effect reported for carbonic anhydrase-arylsulfonamide complexes [10, 11, 6]. In this model, ligand binding reorganizes the hydration network within the active site and releases confined water molecules into the bulk solvent, where they form more favorable hydrogen-bonding interactions. As a result, water reorganization contributes favorably to both enthalpy, through improved hydrogen bonding, and entropy, through increased translational and rotational freedom, leading to co-favorable ΔH and ΔS. The larger enthalpic and entropic contributions together with the more negative ΔC_P observed for PFBS relative to 4FBS suggest that its more highly fluorinated ring induces more extensive restructuring of the active-site hydration network. This interpretation is further supported by recent crystallographic studies showing that the BCA binding pocket contains both hydrophobic and hydrophilic regions, and that it is the rapid rearrangement of active-site water molecules rather than large protein conformational changes that is responsible for the binding process [12]. Accordingly, the observed negative ΔC_P is consistent with binding-induced changes in the hydration network.

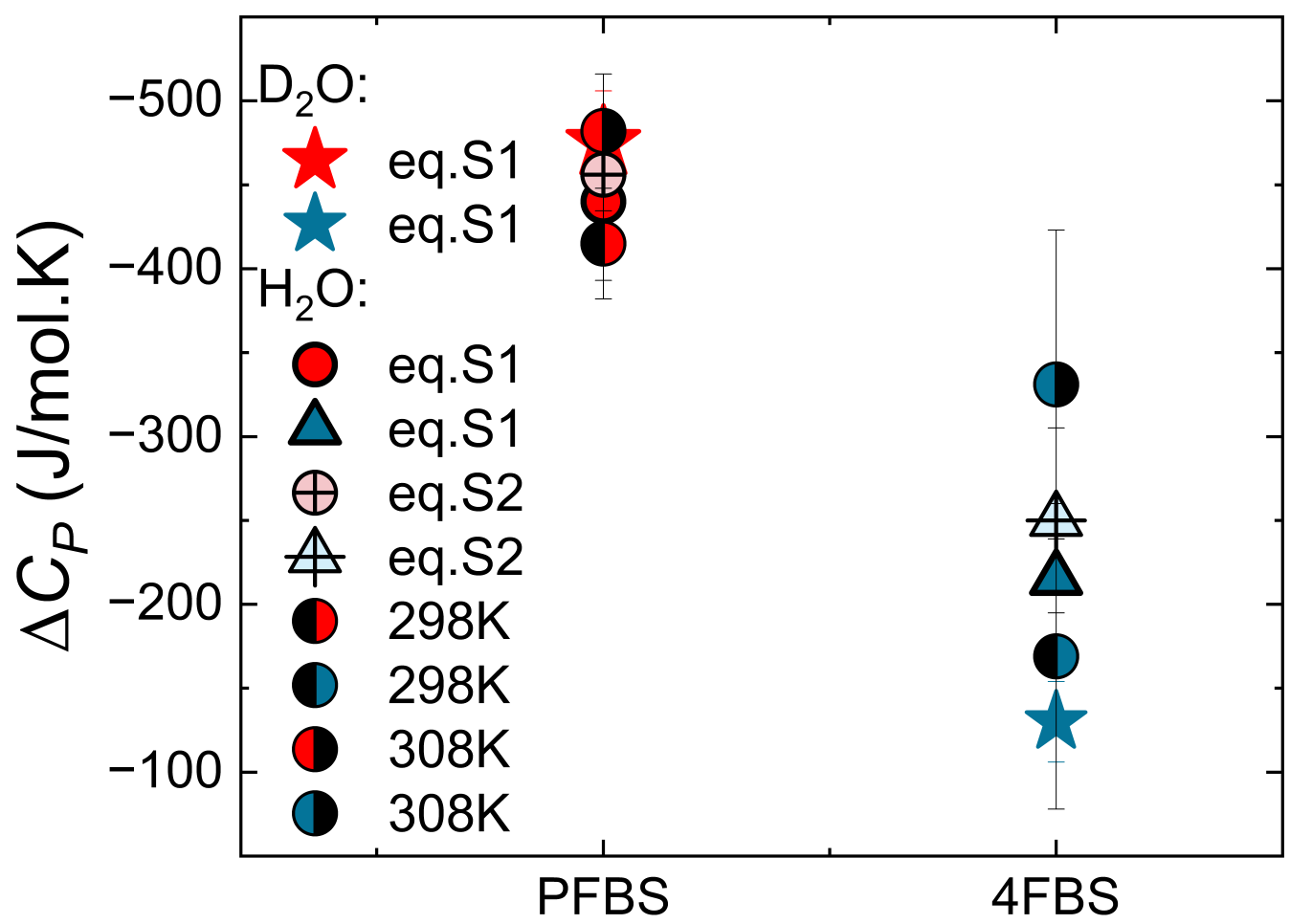

Figure S5: ΔC_P values for PFBS/BCA and 4FBS/BCA in D_2O (star symbols) and H_2O (circle and triangle symbols). The ΔC_P values were calculated using Eqs. S1 and S2, as indicated alongside the corresponding symbols. In addition, ΔC_P was estimated from the local slope of the polynomial fit to the ΔH versus temperature data at specific temperatures. These estimates are represented by the values at 298 K and 308 K, as indicated in the legend.

Both ligands also exhibit a trend toward increasingly negative ΔC_P with increasing temperature (298–308 K), which may reflect enhanced temperature dependent reorganization of the confined hydration network and/or changes in the population of water molecules displaced upon binding. The larger spread in ΔC_P values obtained for 4FBS using eqns. S1, S2, and the polynomial-fit slope at 298 and 308 K indicates greater uncertainty in its ΔC_P estimates compared with PFBS. However, this temperature dependence is not statistically significant and is therefore interpreted cautiously. Additionally, contributions from subtle protein conformational changes cannot be excluded without complementary structural or computational studies.

S7 Calculation of the Ligand Concentration for Complete Protein Binding

In the following, we calculate the ligand concentration required to achieve a high fraction of protein-ligand complexes. The dissociation constant, K_D,represents the ligand concentration at which 50% of the protein is bound, and is defined as:

$$K_D = \frac{[P]\,[L]}{[PL]}, \tag{S3}$$

where $[P]\,,[L]$ and $[PL]$ denote the equilibrium concentrations of free protein, free ligand, and protein-ligand complex, respectively. Let P_0 and L_0 be the initial total concentrations of protein and ligand, and let C be the equilibrium concentration of the complex. Then, the mass balance relations yield:

$$K_D = \frac{(P_0 - C)\,(L_0 - C)}{C} \tag{S4}$$

Solving this quadratic equation for C yields two solutions:

$$C_{\pm} = \frac{K_D + P_0 + L_0}{2} \pm \frac{\sqrt{K_D^2 + 2K_D\,(P_0 + L_0) + (L_0 - P_0)^2}}{2} \tag{S5}$$

The solution with the "+" sign is unphysical, as it results in a complex concentration exceeding the initial protein concentration. Therefore, the physically meaningful solution is $C = C_-$. The fraction of the free protein P_f is given by

$$P_f = \frac{P_0 - C}{P_0} \tag{S6}$$

To determine the required ligand concentration L_0 for a given fraction of free protein, we solve Equation S6 for L_0, which yields:

$$L_0 = \frac{(1 - P_f)(P_f P_0 + K_D)}{P_f} \tag{S7}$$

For example, setting $P_f = 0.001$ (i.e., only 0.1% of the protein remains unbound) allows one to compute the necessary ligand concentration in mol/L. Note that both the protein concentration P_0 and the dissociation constant K_D must be expressed in the same units—typically mol/L—for consistency.

References

[1] Calculator Plugins were used for structure property prediction and calculation, Marvin 24.1.3, 2024, ChemAxon (http://www.chemaxon.com).

[2] V. N. Viswanadhan, A. K. Ghose, G. R. Revankar, and R. K. Robins. Atomic physicochemical parameters for three dimensional structure directed quantitative structure-activity relationships. 4. additional parameters for hydrophobic and dispersive interactions and their application for an automated superposition of certain naturally occurring nucleoside antibiotics. *J. Chem. Inf. Model.*, 29:163–172, 1989.

[3] D. Niether and S. Wiegand. Thermophoresis of biological and biocompatible compounds in aqueous solution. *J. Phys.: Condes. Matter*, 31:503003, 2019.

[4] Vijay M. Krishnamurthy, Brooks R. Bohall, Chu-Young Kim, Demetri T. Moustakas, David W. Christianson, and George M. Whitesides. Thermodynamic parameters for the association of fluorinated benzenesulfonamides with bovine carbonic anhydrase ii. *Chem. Asian J.*, 2:94–105, 2007.

[5] M. Sarter, J. R. Stewart, G. J. Nilsen, M. Devonport, and K. Nemkovski. Data analysis of dynamics in protein solutions using quasi-elastic neutron scattering-important insights from polarized neutrons. *J. Am. Chem. Soc.*, 146(41):28023–28033, 2024.

[6] B. K. Paul. Classical vs. nonclassical hydrophobic interactions underlying various interaction processes: Application of isothermal titration calorimetry. *Chem. Phys. Impact*, 5:100104, 2022.

[7] A. Zubrienė, J. Smirnovienė, A. Smirnov, V. Morkūnaitė, V. Michailovienė, J. Jachno, V. Juozapaitienė, P. Norvaišas, E. Manakova, S. Gražulis, and D. Matulis. Intrinsic thermodynamics of 4-substituted-2,3,5,6-tetrafluorobenzenesulfonamide binding to carbonic anhydrases by isothermal titration calorimetry. *Biophysical Chemistry*, 205:51–65, 2015.

[8] J. M. Sturtevant. Heat capacity and entropy changes in processes involving proteins. *Proc. Natl. Acad. Sci. U.S.A.*, 74(6):2236–2240, 1977.

[9] V. Paketurytė, A. Zubrienė, J. E. Ladbury, and D. Matulis. Intrinsic thermodynamics of protein-ligand binding by isothermal titration calorimetry as aid to drug design. In *in Methods Mol. Biol*, pages 61–74. Springer New York, 2019.

[10] P. W. Snyder, J. Mecinovic, D. T. Moustakas, S. W. Thomas, M. Harder, E. T. Mack, M. R. Lockett, A. Héroux, W. Sherman, and G. M. Whitesides. Mechanism of the hydrophobic effect in the biomolecular recognition of arylsulfonamides by carbonic anhydrase. *Proc. Natl. Acad. Sci. U.S.A.*, 108(44):17889–17894, 2011.

[11] J. Mecinović, P. W. Snyder, K. A. Mirica, S. Bai, E. T. Mack, R. L. Kwant, D. T. Moustakas, A. Héroux, and G. M. Whitesides. Fluoroalkyl and alkyl chains have similar hydrophobicities in binding to the "hydrophobic wall" of carbonic anhydrase. *J. Am. Chem. Soc.*, 133(35):14017–14026, 2011.

[12] J. K. Kim, S. W. Lim, H. Jeong, C. Lee, S. Kim, Dong W. Son, R. Kumar, J. T. Andring, C. Lomelino, J. L. Wierman, A. E. Cohen, T. J. Shin, C. M. Ghim, R. McKenna, B. H. Jo, D. Min, J. M. Choi, and C. U. Kim. Fast product release requires active-site water dynamics in carbonic anhydrase. *Nat. Commun.*, 16:4404, 2025.
